# Supplementary material for: Protein-Binding RNA Aptamers Affect Molecular Interactions Distantly from Their Binding Sites
Source: PLoS One. 2015 Mar 20;10(3):e0119207. doi: 10.1371/journal.pone.0119207 (PMC4368798; doi:10.1371/journal.pone.0119207)
Supplement: S1 Table — (PDF) [file pone.0119207.s001.pdf]

Aptamer	Aptamer sequence
**Upanap-12**	GGGGCCACCAACGACAUUUGCGACUGUUAUAACCUAACAGCGACGUAAAGAUAGUUGAUAUAAAUAGUGCCCAUGGAUC
**Upanap-126**	GGGGCCACCAACGACAUUCAUUCGCACGCUGUGUGGGGAUUAGUCCCGAUGUUGUUGAUAUAAAUAGUGCCCAUGGAUC
**Control RNA**	GGGGCCACCAACGACAUUAACUCACGUUGCAACUAAUACGCUGAGUGGAAACCGUUGAUAUAAAUAGUGCCCAUGGAUC
**Upanap-12.49**	GGACGACAUUUGCGACUGUUAUAACCUAACAGCGACGUAAAGAUAGUCC
**Upanap-12.33**	GGUGCGACUGUUAUAACCUAACAGCGACGUACC
