# Supplementary figures and images for: Protein-Binding RNA Aptamers Affect Molecular Interactions Distantly from Their Binding Sites
Source: PLoS One. 2015 Mar 20;10(3):e0119207. doi: 10.1371/journal.pone.0119207 (PMC4368798; doi:10.1371/journal.pone.0119207)

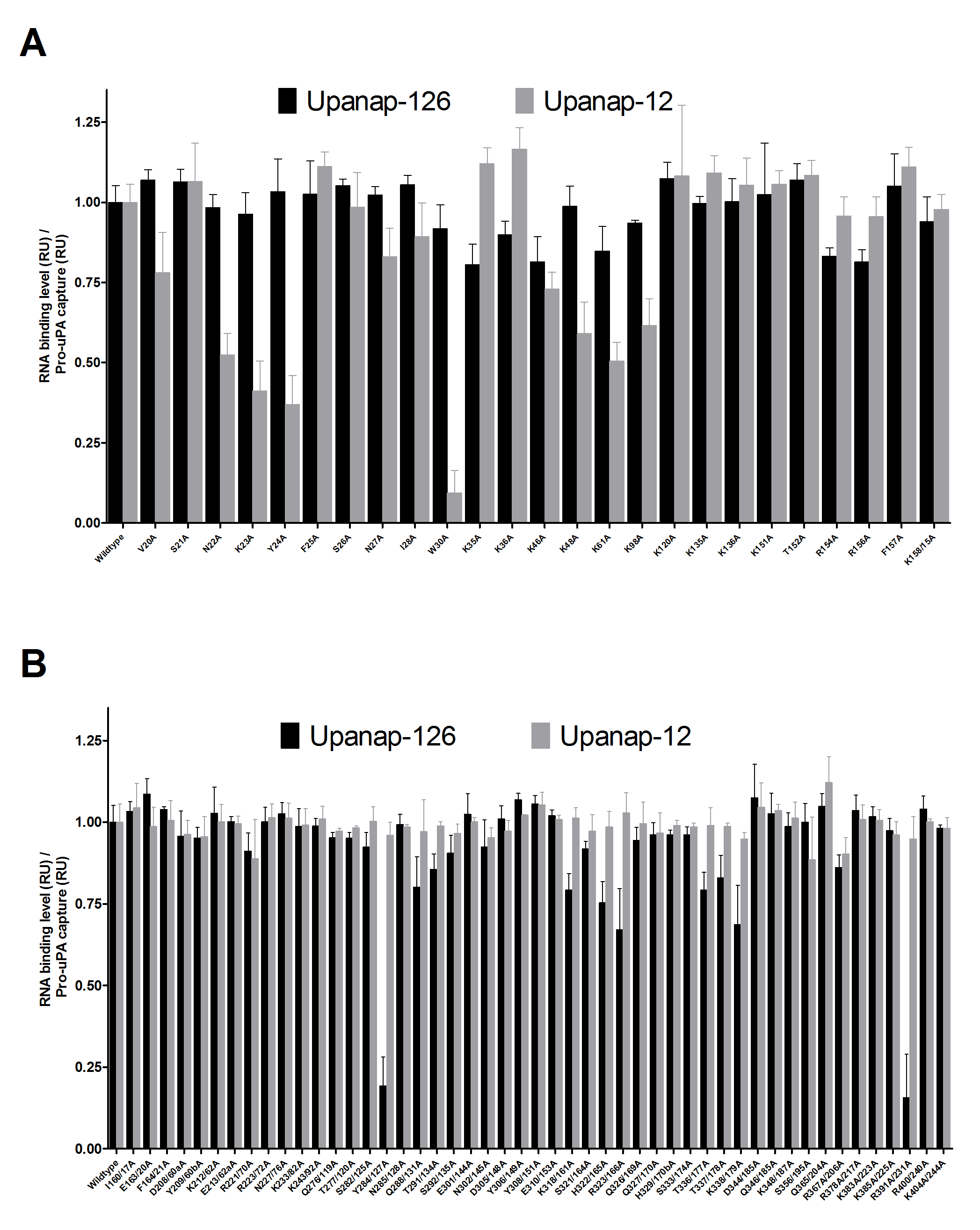

Supplement: S1 Fig — Each of the 74 pro-uPA alanine mutants were captured on a SPR sensor surface carrying immobilized kringle-specific anti-uPA antibody mAb-6 at a level of around 200 RU. The binding level achieved after 60 seconds of association of either 15 nM upanap-126 or upanap-12 was subsequently recorded. For each mutant, the exact number of RU of bound aptamer was divided by the number of RU of captured pro-uPA. The resulting number was normalized against the number for pro-uPA wild type. The figure summarizes the results with A-chain mutants (A) and B-chain mutants (B). (TIF) [file pone.0119207.s002.tif]

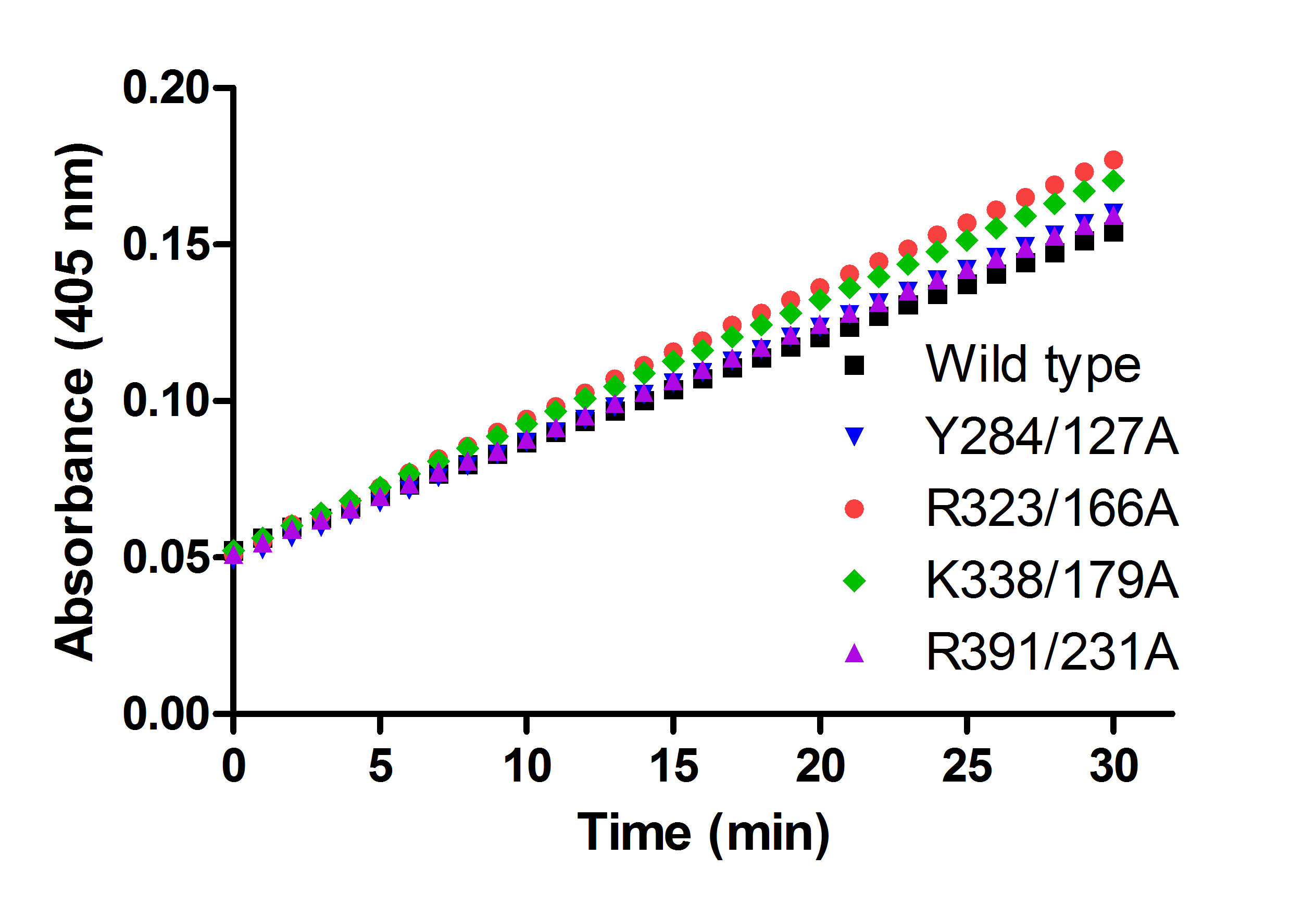

Supplement: S2 Fig — Catalytic activity of 5 nM wild type uPA (black squares) or uPA mutants Y284/127A (blue triangles), R323/166A (red spheres), K338/179A (green diamonds) and R391/231A (purple triangles) towards 250 μM of peptidic chromogenic uPA substrate after complete activation (2 hours with 2.5 nM plasmin). Absorbance at 405 nm was monitored over time and did not indicate any major differences between variants in terms of catalytic activity. The results represent one of three similar independent measurements. (TIF) [file pone.0119207.s003.tif]

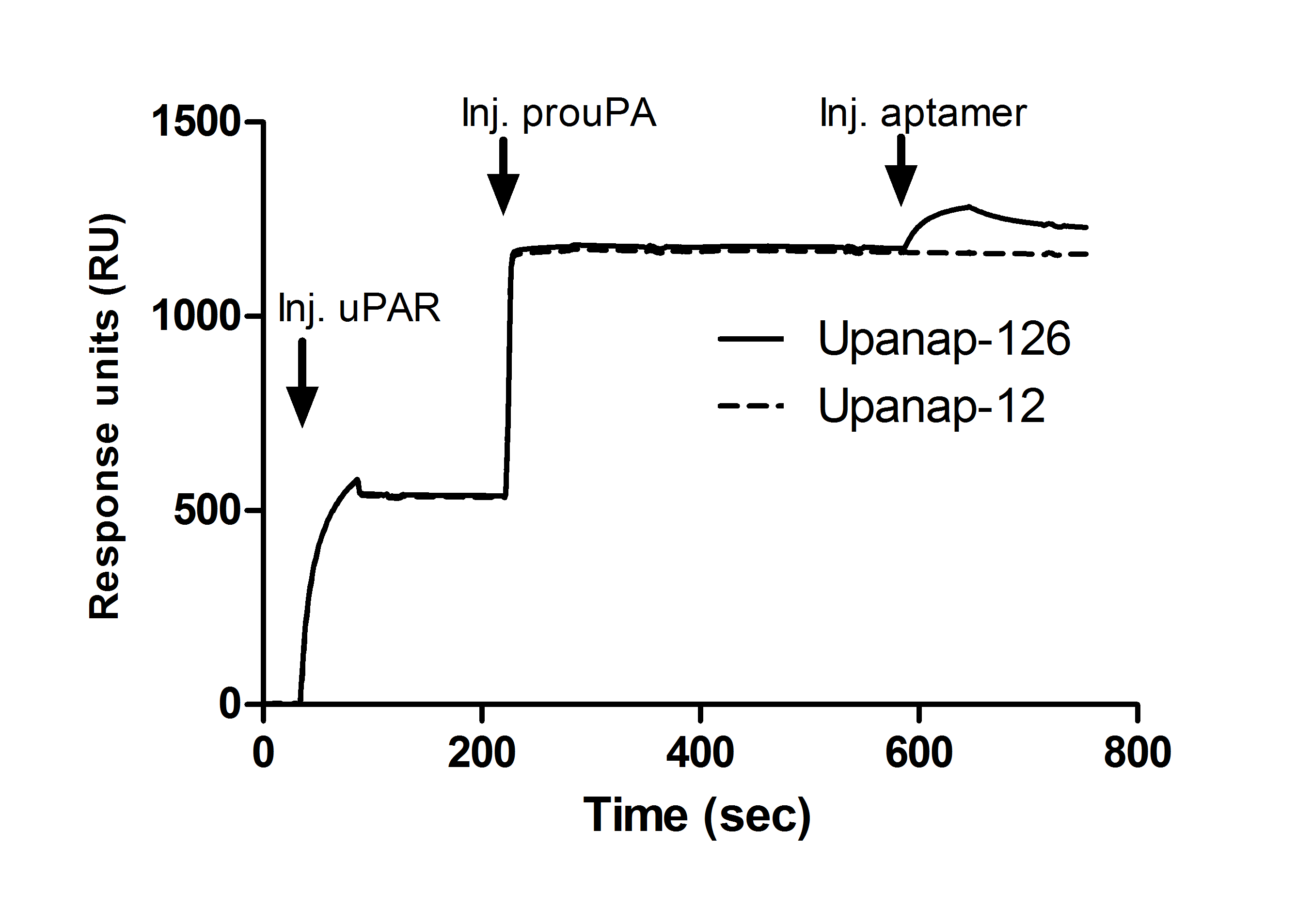

Supplement: S3 Fig — A sensor surface was coupled with anti-uPAR antibody R2. uPAR was subsequently passed over the sensor surface followed by pro-uPA. The association and dissociation of 100 nM upanap-12 (broken line) and upanap-126 (black line) upon injection is shown. (TIF) [file pone.0119207.s004.tif]

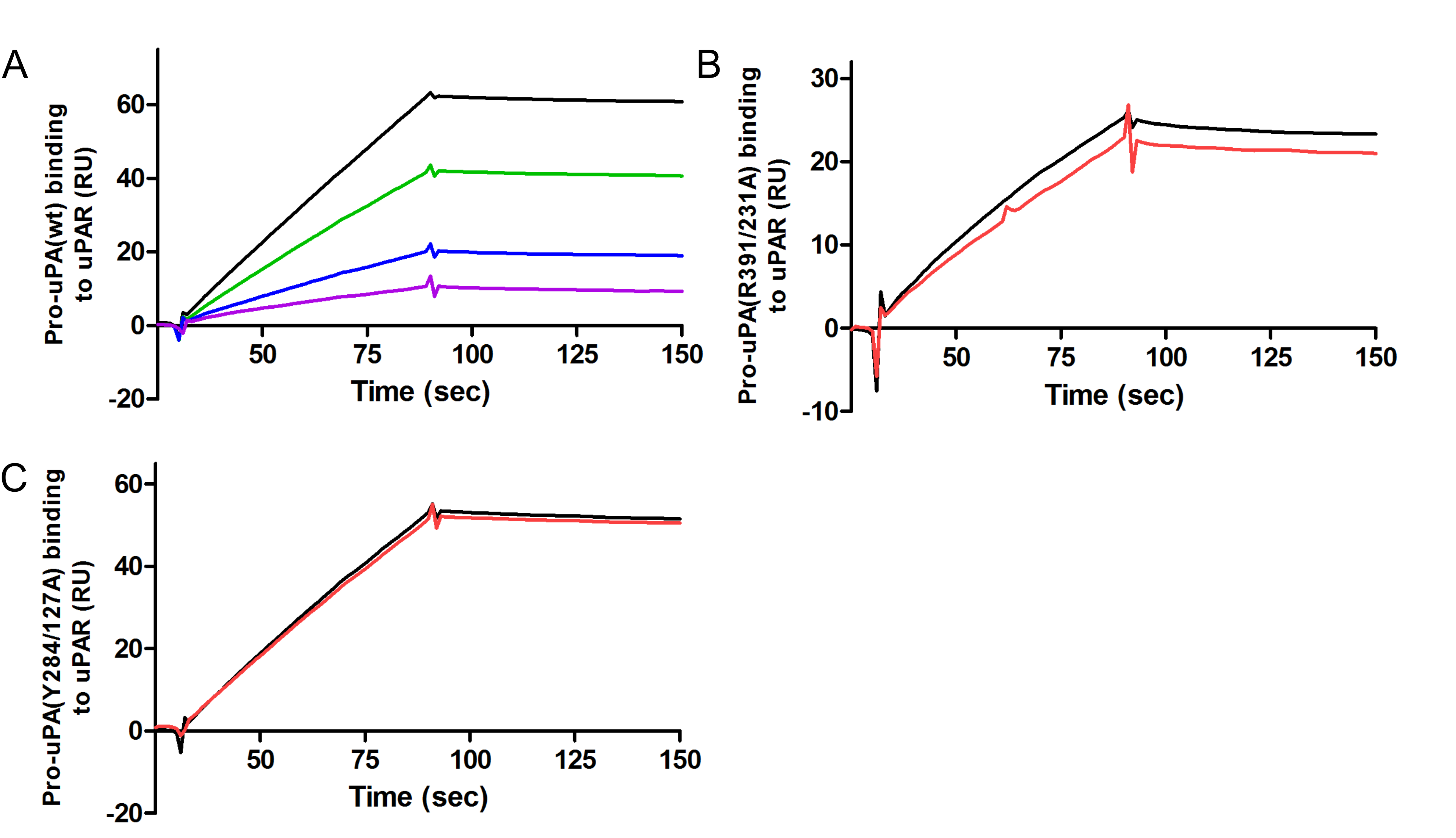

Supplement: S4 Fig — The SPR sensorgrams (A-C) show examples of the association and dissociation phases for binding of 2 nM wt pro-uPA (A) as well as pro-uPA mutants R391/231A (B) or Y284/127A (C) to uPAR on the sensor surface (black lines). In each figure coloured lines represent wild type pro-uPA pre-incubated with either 3.13 nM (green), 12.5 nM (blue) or 50 nM (purple) upanap-126 (A), or pro-uPA mutants pre-incubated with 200 nM (red) upanap-126 (B and C, respectively). (TIF) [file pone.0119207.s005.tif]

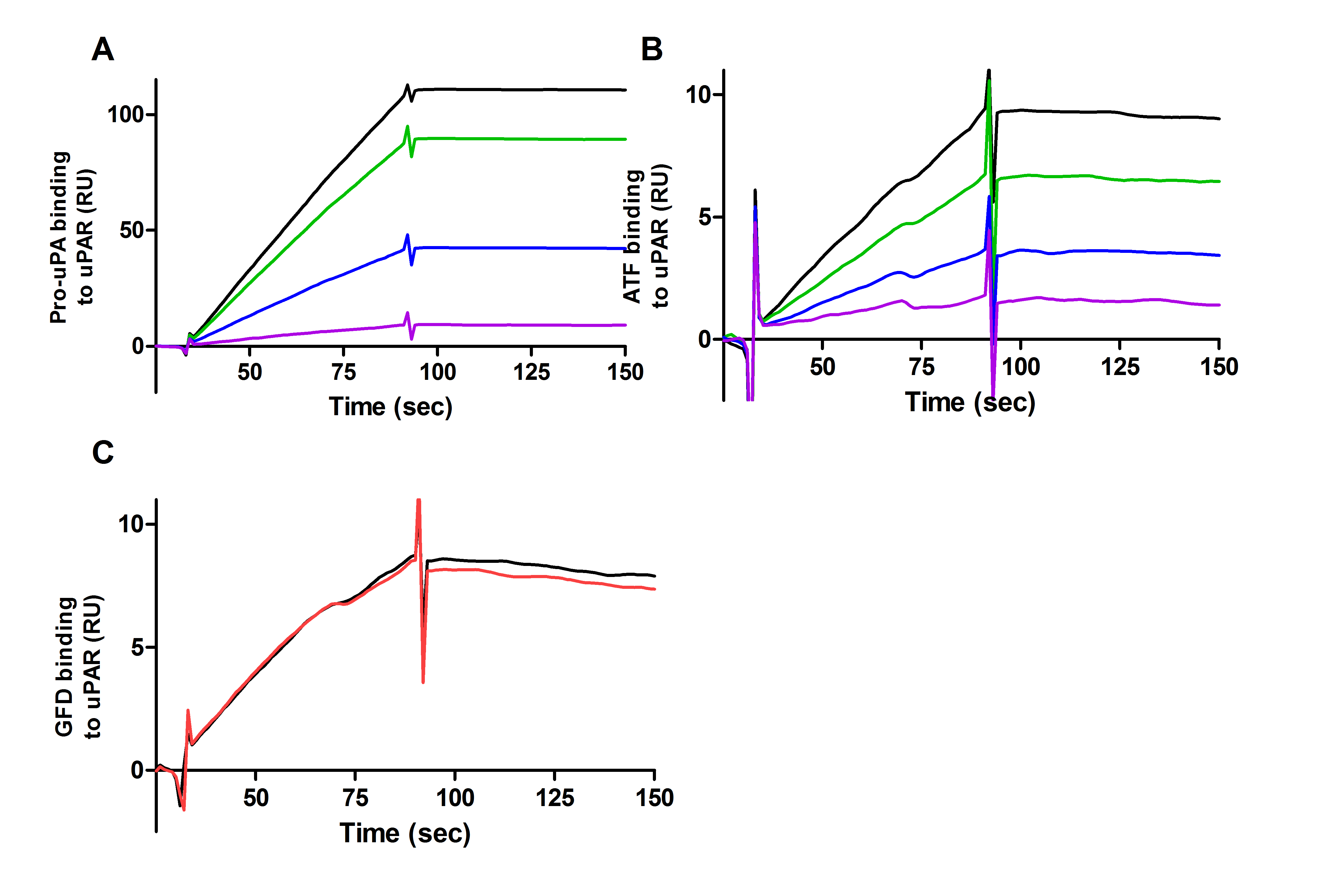

Supplement: S5 Fig — 4 nM of pro-uPA, ATF or GFD was passed over a CM5 surface with immobilized uPAR with or without upanap-12. In A-C the black line represents binding to uPAR of either pro-uPA (A), ATF (B) or GFD (C) alone without RNA. In A and B the coloured lines represent the binding of pro-uPA or ATF to uPAR after pre-incubation with 0.78 nM (green), 3.13 nM (blue) and 12.5 nM (purple) of upanap-12 (). In C, the red line represents binding of GFD to uPAR after pre-incubation with 50 nM of upanap-12. (TIF) [file pone.0119207.s006.tif]

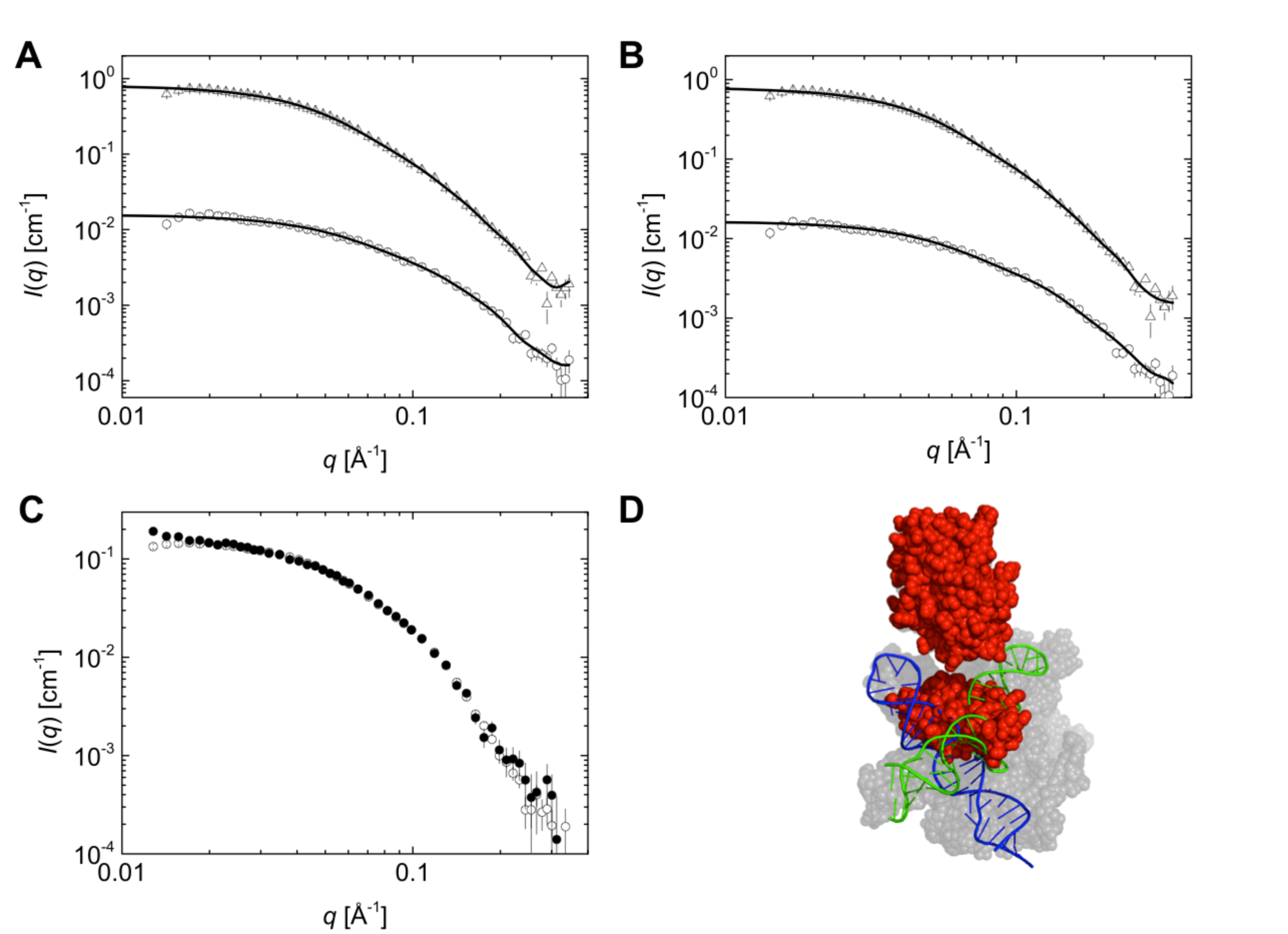

Supplement: S6 Fig — (A) SAXS data obtained for the free upanap-12.49 aptamer (open circles) and the aptamer:pro-uPA complex (open triangels) with their corresponding IFT model fits (black line). (B) SAXS data obtained for free upanap-12.49 (open circles) and upanap-12.49:pro-uPA complex (open triangels) with their corresponding model fits (black line) for the most representative ab initio model. The SAXS data for the complex in panel A and B is rescaled with a scale factor of 10 to improve visualization of the data. (C) SAXS data obtained for pro-uPA in the current study (closed circles) compared to pro-uPA from a previous study [25] (open circles). (D) Rigid-body models for the upanap-12.49:pro-uPA complex with the full-length pro-uPA structural model in red and the two most representative aptamer12.49 models in green and blue. The semitransparent beads represent the ab initio space of the complex after subtraction of the ab initio space of uPA, illustrating the residual space for the RNA to occupy. (TIF) [file pone.0119207.s007.tif]
