# Supplementary material for: Correction: Protein-Binding RNA Aptamers Affect Molecular Interactions Distantly from Their Binding Sites
Source: PLoS One. 2015 Apr 17;10(4):e0126782. doi: 10.1371/journal.pone.0126782 (PMC4401791; doi:10.1371/journal.pone.0126782)
Supplement: S1 File — (PDF) [file pone.0126782.s001.pdf]

PLOS ONE

RESEARCH ARTICLE

Protein-Binding RNA Aptamers Affect Molecular Interactions Distantly from Their Binding Sites

Daniel M. Dupont[1,2]*, Cathrine K. Thuesen[1,2], Kenneth A. Bøtkjær[1,2], Manja A. Behrens[3,5], Karen Dam[1], Hans P. Sørensen[1,2], Jan S. Pedersen[3], Michael Ploug[2,4], Jan K. Jensen[1,2], Peter A. Andreasen[1,2]

1 Department of Molecular Biology and Genetics, Aarhus University, Aarhus, Denmark, **2** Danish-Chinese Centre for Proteases and Cancer, Aarhus University, Aarhus, Denmark, **3** iNANO Interdisciplinary Nanoscience Center and Department of Chemistry, Aarhus University, Aarhus, Denmark, **4** Finsen Laboratory, Rigshospitalet and Biotech Research & Innovation Centre, Copenhagen, Denmark, **5** Department of Chemistry, Lund University, Lund, Sweden

* dmd@mbg.au.dk

CrossMark
click for updates

OPEN ACCESS

Citation: Dupont DM, Thuesen CK, Bøtkjær KA, Behrens MA, Dam K, Sørensen HP, et al. (2015) Protein-Binding RNA Aptamers Affect Molecular Interactions Distantly from Their Binding Sites. PLoS ONE 10(3): e0119207. doi:10.1371/journal.pone.0119207

Academic Editor: Henning Ulrich, University of São Paulo, BRAZIL

Received: October 8, 2014

Accepted: January 11, 2015

Published: March 20, 2015

Copyright: © 2015 Dupont et al. This is an open access article distributed under the terms of the Creative Commons Attribution License, which permits unrestricted use, distribution, and reproduction in any medium, provided the original author and source are credited.

Data Availability Statement: All relevant data are within the paper and its Supporting Information files.

Funding: This work was supported by grants from the Danish Cancer Society [R40-A1838 to KAB, R56-A2997-12-S2]; the Carlsberg Foundation [2010-01-0819 to DMD]; the Danish National Research Foundation [26-331-6]; the Danish Research Agency [272-06-0518]; and the Novo Nordisk Foundation [R114-A11382]. The funders had no role in study design, data collection and analysis, decision to publish, or preparation of the manuscript.

Abstract

Nucleic acid aptamer selection is a powerful strategy for the development of regulatory agents for molecular intervention. Accordingly, aptamers have proven their diligence in the intervention with serine protease activities, which play important roles in physiology and pathophysiology. Nonetheless, there are only a few studies on the molecular basis underlying aptamer-protease interactions and the associated mechanisms of inhibition. In the present study, we use site-directed mutagenesis to delineate the binding sites of two 2´-fluoropyrimidine RNA aptamers (upanap-12 and upanap-126) with therapeutic potential, both binding to the serine protease urokinase-type plasminogen activator (uPA). We determine the subsequent impact of aptamer binding on the well-established molecular interactions (plasmin, PAI-1, uPAR, and LRP-1A) controlling uPA activities. One of the aptamers (upanap-126) binds to the area around the C-terminal α-helix in pro-uPA, while the other aptamer (upanap-12) binds to both the β-hairpin of the growth factor domain and the kringle domain of uPA. Based on the mapping studies, combined with data from small-angle X-ray scattering analysis, we construct a model for the upanap-12:pro-uPA complex. The results suggest and highlight that the size and shape of an aptamer as well as the domain organization of a multi-domain protein such as uPA, may provide the basis for extensive sterical interference with protein ligand interactions considered distant from the aptamer binding site.

Introduction

The SELEX procedure (systematic evolution of ligands by exponential enrichment) allows the screening of large random-sequence oligonucleotide (RNA/DNA) libraries for sequences capable of binding to a protein target of interest [1, 2]. The protein-binding sequences isolated are called aptamers. In many respects they resemble antibodies, *i.e.* they often bind their targets

Competing Interests: The authors have declared that no competing interests exist.

with high affinity and specificity as well as modulate target functions [3, 4]. However, aptamers differ from antibodies in other respects, *e.g.* in terms of their pharmacokinetic and immunogenic profile and in the possibility of producing and modifying them by chemical synthesis. Hence, aptamers are interesting alternatives or supplements to small molecules, peptides and antibodies for use as artificial protein ligands for therapeutic strategies and prototype drugs, and for analytical applications such as imaging and diagnostics.

Many pathological conditions have been linked to dysfunction or dysregulation of proteases. Proteases are therefore often recognized as potential therapeutic targets or prognostic markers [5]. Thrombin was the first protease for which aptamers were described [6]. Since then, more than 40 aptamer selections alone using proteases as targets have been published [3]. Still, most of our detailed understanding of aptamer-target interactions, inhibitory functions and relative sizes of aptamers and their targets comes from studies with a select number of substantially truncated thrombin aptamers [3, 7]. However, aptamers can rarely be reduced in this degree and are therefore often much larger molecules. More studies are therefore needed in order to obtain a more broad molecular understanding of how aptamers bind and affect their target proteins.

The urokinase-type plasminogen activator, uPA, is an M_r ~50,000 modular serine protease consisting of an N-terminal epidermal growth factor-like domain (GFD, residues 1–48) and a kringle domain (KD, residues 49–131), collectively known as the amino-terminal fragment (ATF), followed by a C-terminal catalytic serine protease domain (residues 148/1–411/251) [8, 9]. For the serine protease domain of human uPA, a double numbering system is used, the first number starting from the N-terminus of uPA, the second number corresponding to the chymotrypsinogen template numbering system. The catalytic domain is tethered to the kringle domain by a 16 amino acid linker sequence. uPA is secreted from cells as an inactive zymogen (pro-uPA) that can be activated by proteolytic cleavage of a single peptide bond (K158/15-I159/16). The resulting A-chain (residues 1–158) and B-chain (159/16–411/251) are covalently linked by a disulfide bridge between cysteines 148/1 and 279/122. Pro-uPA as well as active uPA can bind the uPA receptor, uPAR, on the cell surface. Receptor binding is mediated by the β-hairpin (residues 19–31) of the GFD. Here, trace amounts of plasmin is thought to initiate pro-uPA activation, which in turn activates more plasminogen. This arrangement provides the cell with a controlled proteolytic potential towards extracellular matrix (ECM) proteins, which are being turned over during cell migration and invasion events. In addition, pro-uPA binding to uPAR activates the adhesive and cell signaling functions of uPAR, including the interaction of uPAR with the somatomedin B domain (SMB) of the ECM protein vitronectin (VN) [8, 10]. The uPA proteolytic activity is regulated by the serpin plasminogen activator inhibitor-1 (PAI-1) [11]. The covalently linked uPA:PAI-1 inhibitory complex is cleared from the cell surface by endocytosis receptors, such as the low density lipoprotein receptor-related protein-1A (LRP-1A) [11, 12]. uPA participates in many events of tissue remodeling in the healthy organism, but is also known to be a prognostic marker in cancer and to mediate cancer metastasis [8, 9, 13]. uPA is therefore a potential target for anti-cancer therapy.

We have previously isolated two different nuclease-resistant 2'-fluoropyrimidine-modified (2'-F-Y) RNA aptamers binding to human uPA. One of them, upanap-12, appears to bind the ATF and to be a potent inhibitor of the binding of uPA to uPAR [14]. This aptamer is currently the only aptamer known to bind to a non-catalytic domain of a serine protease. The other aptamer, upanap-126, was selected against the zymogen form of the catalytic domain, but also binds active uPA [15]. Upanap-126 is a multi-functional inhibitor of uPA, inhibiting the activation of pro-uPA, uPA binding to uPAR, as well as binding of the uPA:uPAR complex to vitronectin. In addition, upanap-126 was found to inhibit invasion and dissemination of cancer cells in simple *in vivo* chicken models of tumor dissemination [15]. Both aptamers exploit

alternative strategies for inhibiting uPA activities as compared with the more classical approach for inhibiting serine protease activity by targeting the active site. We therefore reasoned that further analysis of structure-function relationships of the aptamers could be informative about the mechanisms by which such RNA aptamers affect the molecular interactions and functions of proteins in general and proteases in particular.

In the present study, we focused on the abilities of the aptamers to interfere with the interactions between uPA and its physiological ligands, substrates and processing enzymes. We find that both aptamers exhibit extensive pleiotropic inhibitory profiles. Accompanied by binding site analysis using site-directed mutagenesis and small-angle X-ray scattering (SAXS), we advance a molecular explanation for the diverse functional properties and action of these two potential therapeutic aptamers.

Results

Binding site of upanap-126 on the catalytic domain of uPA

To delineate the binding site of upanap-126 on pro-uPA, we analyzed 74 mutants with single-site alanine replacements of surface exposed residues (25 in the A-chain and 49 in the B-chain) by surface plasmon resonance (SPR) binding analysis. Alanine mutation was performed in various regions of uPA, including in particular the area of the active site, the pro-uPA activation site and the uPAR binding site due to the inhibitory properties of the aptamers. After capturing comparable levels of the pro-uPA mutants on an immobilized anti-uPA antibody (mAb-6), binding levels achieved by subsequent injections of 15 nM upanap-126 were recorded. The obtained levels of aptamer binding were calculated relative to the amount of captured pro-uPA. From all 74 mutants analyzed (S1A Fig. for A-chain mutants; supplementary S1B Fig. for B-chain mutants), a few hotspot residues were identified (Fig. 1A). Two mutations in particular (Y284/127A and R391/231A) had a major impact on upanap-126 binding to pro-uPA (>50%). They are located near the N-terminus of the C-terminal helix in the catalytic domain of pro-uPA, almost on the back relative to the active site (Fig. 2). Two additional mutants (R323/166A and K338/179A) located in this region exhibited a more moderate impact on uPA binding (25–50%) as shown in Fig. 1A. To ensure that mutations did not affect the overall structure and function of the protease domain, we measured the catalytic activity of the mutants after activation by plasmin and did not observe any major differences compared with wild type pro-uPA (S2 Fig.).

Binding site for upanap-12 in the ATF of uPA

The 74 mutants were also screened by SPR analysis for mutations affecting the binding of the ATF-binding aptamer upanap-12 to pro-uPA. This analysis revealed the importance of both the β-hairpin of the GFD as well as the kringle domain (Figs. 1B and S1A), whereas none of the mutations to the catalytic domain affected binding (Figs. 1A and S1B). The most pronounced effects in aptamer binding were observed for K23A, Y24A and W30A within the β-hairpin (>50%), but moderate effects were also noted for N22A in the β-hairpin and K46A, K48A, K61A, and K98A in the kringle domain (25–50%). The identified β-hairpin residues are all important for uPAR binding and/or uPA-uPAR species specificity, whereas alanine substitution of kringle lysine residues do not affect the interaction [19–21]. The location of these mutations in the ATF structure is shown in Fig. 2, clearly highlighting the composite nature of the binding site where 5 proximate lysine residues form a charged perimeter around the aromatic hotspot residue Trp30.

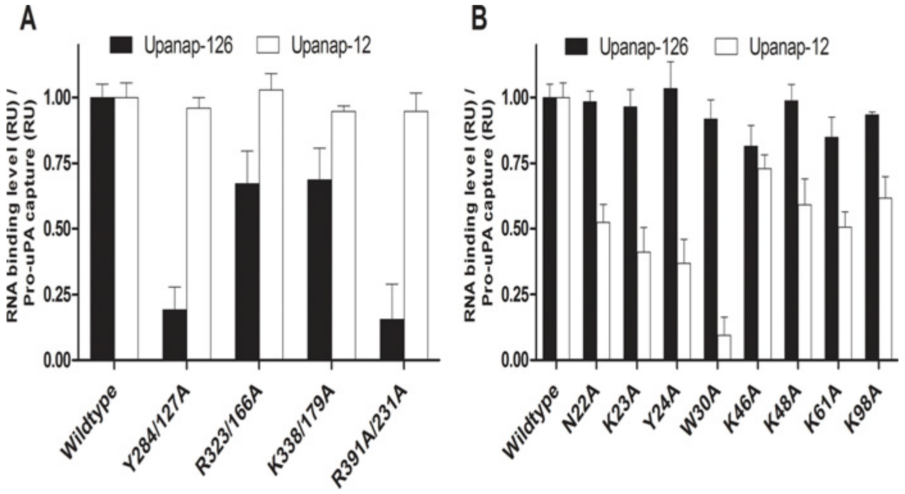

Fig 1. SPR analysis of aptamer binding to pro-uPA mutants. Pro-uPA alanine mutants were captured on a SPR sensor surface carrying the immobilized kringle specific anti-uPA antibody mAb-6 to a level of around 200 RU. The binding level observed for either 15 nM upanap-126 or upanap-12 was subsequently recorded. The exact number of mole aptamer bound per mole captured pro-uPA was calculated for each mutant. The figure summarizes the results with mutants for which a major (>50%) or moderate (25–50%) reduction in binding was observed relative to wild type pro-uPA binding in the case of mutations in (A) the catalytic domain, or (B) the ATF. Each mean value and standard deviation is based on 5 determinations. The entire set of mutants analyzed can be found in S1A Fig. (A-chain mutants) and S1B Fig. (B-chain mutants).

doi:10.1371/journal.pone.0119207.g001

The ATF-binding aptamer as well as the catalytic domain-binding aptamer inhibit pro-uPA activation

While upanap-126 has previously been found to be an efficient inhibitor of pro-uPA activation by plasmin [15], the ATF-binding aptamer upanap-12 has not been investigated in this respect. We therefore studied the ability of upanap-12 to inhibit plasmin-catalyzed pro-uPA activation by monitoring the relative rate of hydrolysis of a small peptidic chromogenic uPA substrate (V_i/V_0), as a measure of the amount of active enzyme generated during incubation with plasmin. Like upanap-126 ($IC_{50} = 7.1 \pm 1.6$ nM), upanap-12 was also found to be a potent inhibitor of pro-uPA activation in this assay ($IC_{50} = 3.1 \pm 0.3$ nM; Fig. 3A). We also examined the effect on pro-uPA activation with truncated versions of the ATF-binding aptamer upanap-12 (comprising 79 nucleotides). The truncation variants upanap-12.49 (49 nt) and upanap-12.33 (33 nt) contain the expected important sequence features of upanap-12 and were previously found to interfere with pro-uPA—uPAR interaction, with similar IC_{50} values comparable to that of the full-length version [14]. We did not observe any detectable differences between inhibitory activities for upanap-12.49 ($IC_{50} = 3.2 \pm 0.3$ nM), upanap-12.33 ($IC_{50} = 5.5 \pm 2.2$ nM) and that of full-length upanap-12 (Fig. 3A).

Upanap-12 interference with plasmin-catalyzed pro-uPA activation was confirmed by immunoblotting analysis of the temporal progression of cleavage of one-chain pro-uPA to two-chain uPA (Fig. 4). In this analysis, upanap-12, as well as upanap-126, delayed the generation of the two-chain form of uPA, while control RNA did not. The truncated variants of upanap-12 inhibited the conversion as efficient as the parent aptamer (Fig. 4).

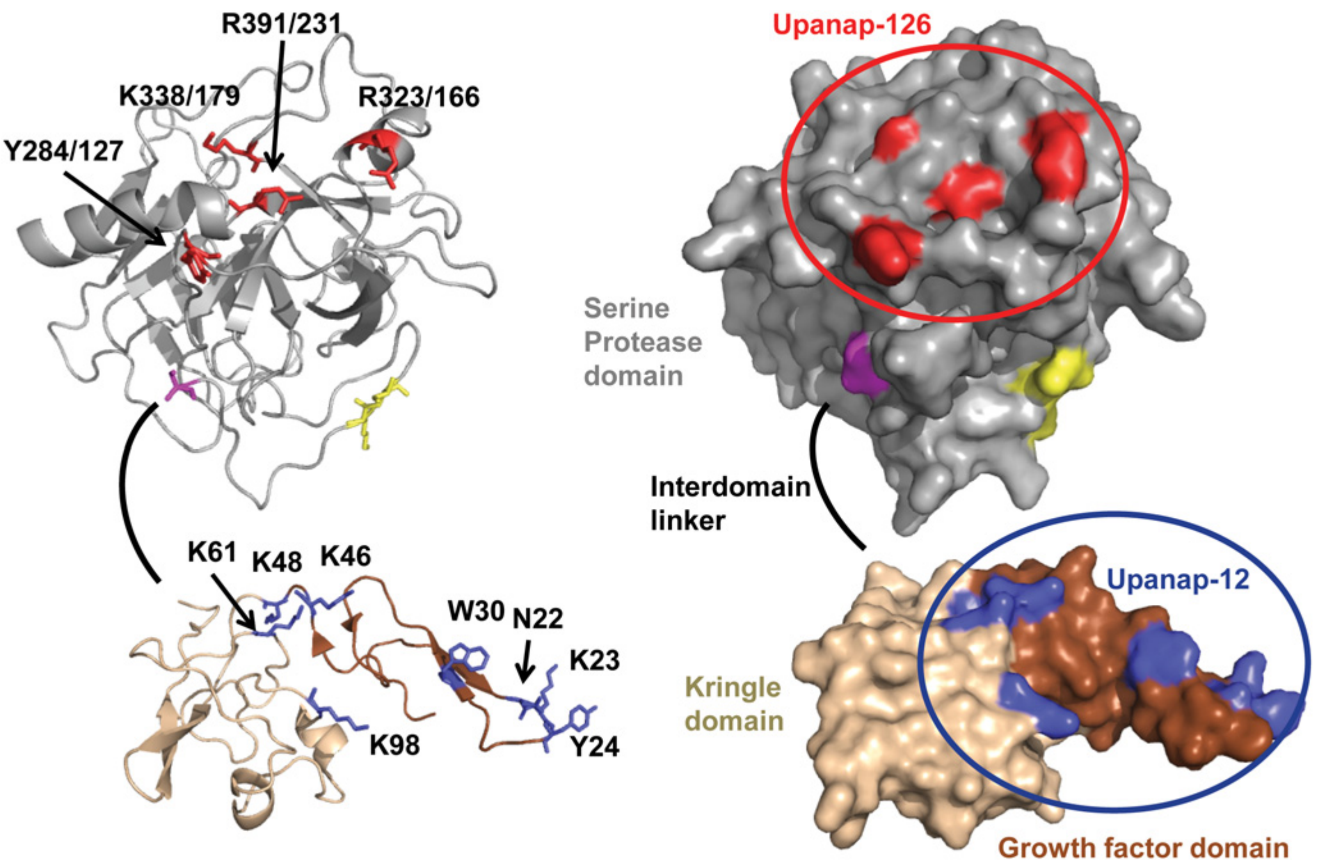

Fig 2. Aptamer binding sites displayed on the three-dimensional structure of pro-uPA in cartoon (left) and surface (right) presentation. The structure of the catalytic domain (gray, residues 148/1–406/246) is a homology model [16] created using the sequence of pro-uPA and the chymotrypsinogen structure (PDB ID 1EX3) [17]; the orientation of individual amino acid residues may not be correct. The activation bond (K158-I159/16) is coloured yellow. Cysteine 148/1 is coloured magenta. The linker between the kringle and the catalytic domain (residues 132–147) is shown as a black line, as its structure is unknown. The structural depiction of the ATF was generated from an existing structure (PDB ID 2I9A) [18]. The growth factor domain (11–48) is coloured brown and the kringle domain (49–131) is coloured wheat. Residues implicated in the binding of upanap-126 are coloured red. Residues implicated in the binding of upanap-12 are coloured blue. The figure was created using PyMOL Viewer.

doi:10.1371/journal.pone.0119207.g002

We then investigated whether the plasmin-catalyzed activation of mutants of pro-uPA could be inhibited by the aptamers using the peptide substrate hydrolysis assay. While the catalytic domain-binding aptamer upanap-126 inhibited zymogen activation for W30A efficiently, the ATF-binding aptamer upanap-12 could not (Fig. 3B and 3C). This result confirms the importance of residue Trp30 for the binding of upanap-12 to pro-uPA. Conversely, and in agreement with the results of the SPR binding site analysis, upanap-12, but not upanap-126, could inhibit plasmin-mediated activation of pro-uPA with the mutations Y284/127A and R391/231A in the catalytic domain (Fig. 3B and 3C).

Finally, we examined if the two uPA aptamers could inhibit plasmin-catalyzed activation of uPAR-bound pro-uPA. Pro-uPA was pre-incubated with a 5-fold molar excess of uPAR at a concentration around 100-fold above the K_D for the uPA—uPAR interaction and then incubated with plasmin in the presence of varying concentrations of aptamer. The hydrolysis of a small peptidic uPA substrate was measured after the incubation. Only upanap-126 (IC_{50} = 27.4 ± 5.2 nM), but not upanap-12, was able to inhibit pro-uPA activation (Fig. 3D). This

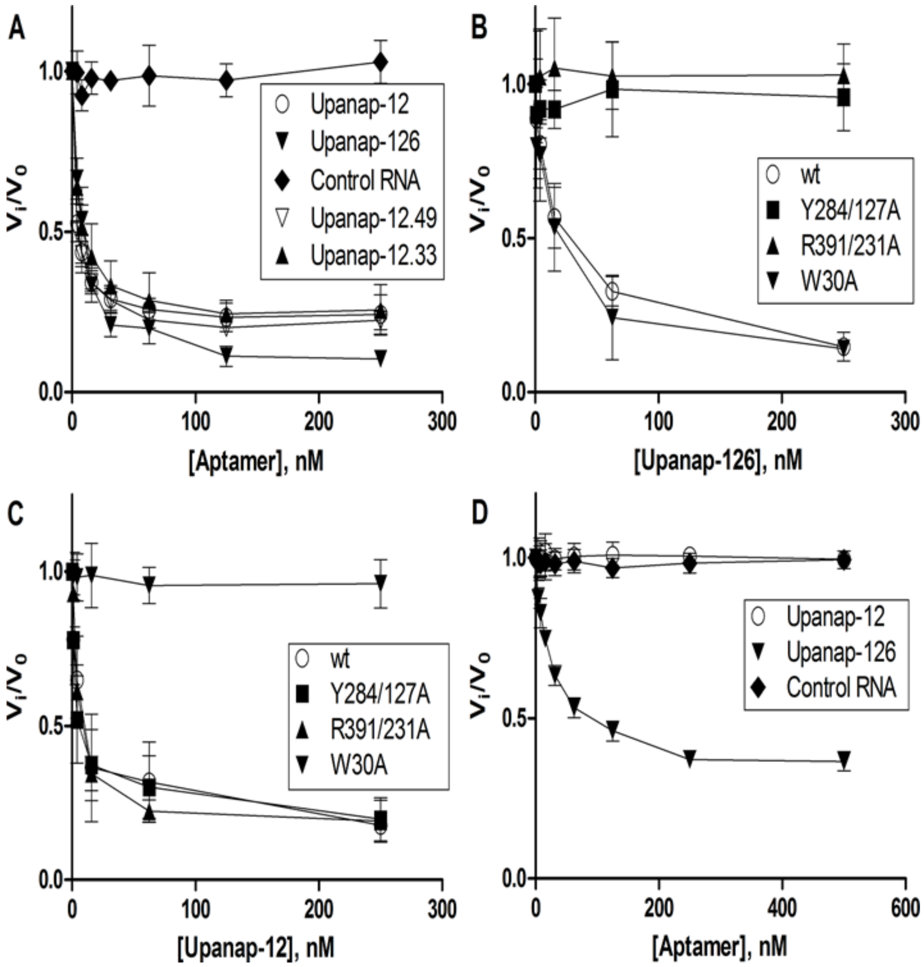

Fig 3. Aptamer interference with plasmin-catalyzed pro-uPA activation. Pro-uPA wild type with and without uPAR (**A** and **D**) or mutants (**B** and **C**) were pre-incubated with aptamer and zymogen activation by plasmin allowed for 30 min. The extent of uPA generation was then estimated from the rate of uPA-catalyzed cleavage of a chromogenic substrate. The graphs show the relative rates of substrate cleavage at a given aptamer concentration as a fraction of controls without aptamers (V_i/V_0). Data represent the average of three independent determinations.

doi:10.1371/journal.pone.0119207.g003

finding is in agreement with the observation that upanap-126, and not upanap-12, is able to bind to uPAR-bound pro-uPA as assessed by surface plasmon resonance (S3 Fig).

Effects of the aptamers on the pro-uPA—uPAR interaction

Both aptamers are low-nanomolar inhibitors of pro-uPA binding to uPAR immobilized on the surface of a SPR sensor surface (Fig. 5A and 5B). For upanap-126, this observation was unexpected, since the aptamer binds to the catalytic domain. We therefore investigated the ability of upanap-126 to inhibit uPAR binding using the pro-uPA mutants Y284/127A and R391/231A. We observed no detectable inhibition in either case (Fig. 5A; sensorgrams in S4 Fig.). In contrast, these mutations did not significantly affect the ability of the ATF aptamer upanap-12 to inhibit pro-uPA binding to uPAR (data not shown). Hence, the inhibitory activity of upanap-126 towards uPA—uPAR binding is dependent on its interaction with the catalytic domain.

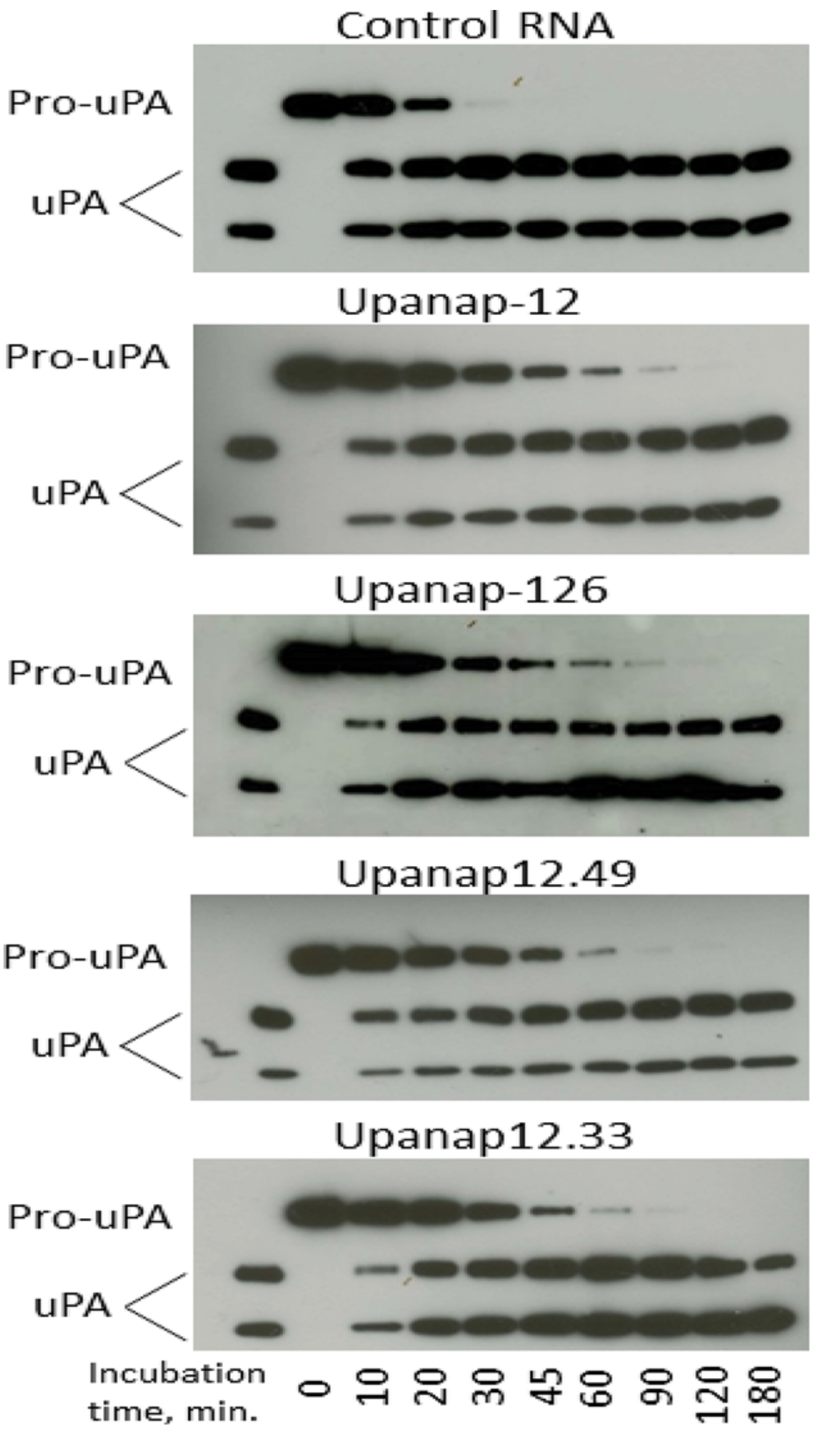

Fig 4. Immunoblotting analysis of the effect of the aptamers on the conversion of one-chain pro-uPA to two-chain uPA. Pro-uPA and plasmin were incubated for the indicated time periods with the indicated aptamers, after which plasmin activity was stopped with HCl. The samples were analyzed by reducing SDS-PAGE and immunoblotting with a polyclonal anti-uPA antibody. Two-chain uPA alone is shown to the left.

doi:10.1371/journal.pone.0119207.g004

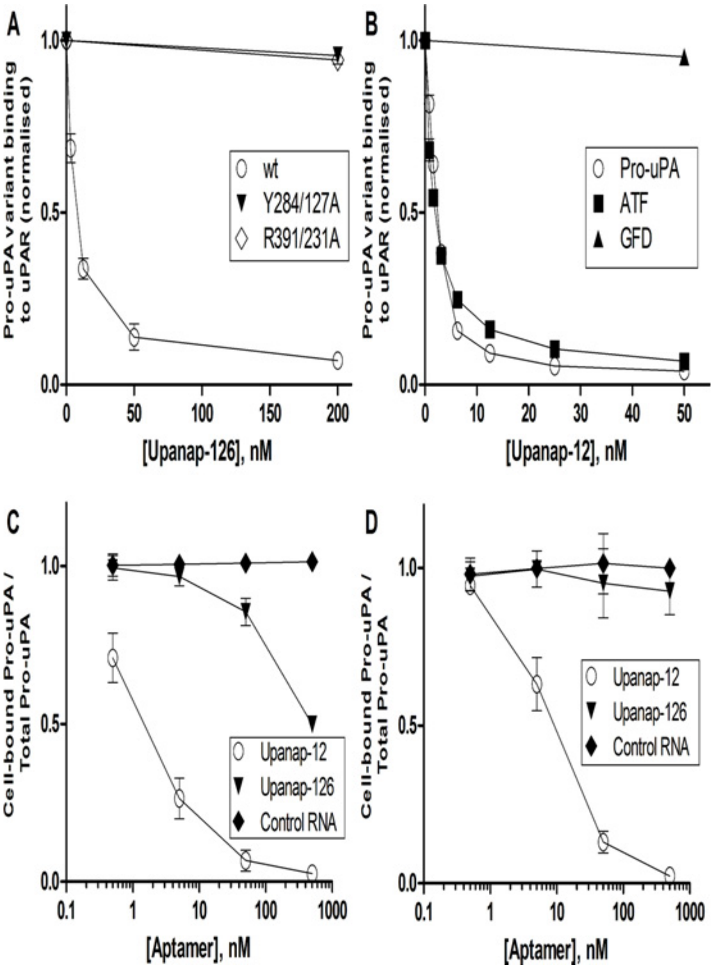

Fig 5. Aptamer inhibition of the binding of uPA to uPAR. (**A**) The binding of pro-uPA (wild type or mutant) to uPAR immobilized on a SPR sensor surface in the presence of the indicated concentrations of upanap-126 was estimated and expressed as a fraction of the binding in the absence of aptamer. Raw data are shown in S4 Fig. With pro-uPA mutants only the effect of the highest dose of upanap-126 (200 nM) was tested. (**B**) The binding of pro-uPA, ATF or GFD to uPAR immobilized on a SPR sensor surface in the presence of the indicated concentrations of upanap-12 was estimated and expressed relative to the binding in the absence of aptamer. Raw data are shown in S5 Fig. With the GFD only the effect of the highest dose of upanap-12 (50 nM) was tested. (**C**) and (**D**) One million U937 cells were incubated with 10 pM ^{125}I-pro-uPA and 0–500 nM upanap-126, upanap-12 or control RNA for either 1 hour (**C**) or 24 hours (**D**), respectively, at 4°C. For each sample, the amount of ^{125}I-pro-uPA bound to the cells was divided by the total amount of ^{125}I-pro-uPA (pellet and supernatant) and normalized to the number obtained for cells without RNA. Data represent the average of three replicates.

doi:10.1371/journal.pone.0119207.g005

We also used the uPAR-coupled sensor surface to confirm the binding site of the ATF aptamer for pro-uPA. Previously, we were unable to detect binding of upanap-12 to a uPA variant lacking the GFD, demonstrating that this domain is necessary for binding [14]. To reconcile this finding with our present implication of kringle domain residues in the binding site of upanap-12 (see above), we measured the effect of the aptamer on the binding of full-length pro-uPA, ATF and GFD to uPAR. Upanap-12 was found to inhibit the binding of pro-uPA and ATF to uPAR with similar IC_{50} values (2.2 ± 0.1 nM and 1.8 ± 0.2 nM, respectively), but unable to inhibit the binding of the GFD alone to uPAR (Fig. 5B; sensorgrams in S5 Fig.). These results

clearly emphasize the importance of an intact ATF for the binding of the ATF-binding aptamer upanap-12 to pro-uPA.

The effect of the aptamers on the uPA—uPAR interaction on live cells was investigated by measuring the amount of ^{125}I-pro-uPA bound to U937 cells after incubations with and without aptamers for various periods of time. Both aptamers were able to interfere with the association of ^{125}I-pro-uPA with the cells during a one-hour incubation (Fig. 5C). Nonetheless, only the ATF-binding aptamer upanap-12 exhibited high efficacy with a (IC_{50} = 1–2 nM). Furthermore, the weaker effect exhibited by upanap-126 (IC_{50} = ~500 nM) did not persist after prolonged incubation at 4°C (Fig. 5D). A separate set of experiments showed that the observed difference between the two aptamers in this assay did not reflect a difference in stability under the assay conditions (data not shown). Therefore, during prolonged exposure of aptamer:pro-uPA complexes to uPAR, the ATF-binding aptamer more efficiently interferes with uPA—uPAR binding. This is in agreement with the observation that upanap-126 can bind concomitantly with uPAR (S3 Fig.), and instead of blocking binding, it may merely reduce the rate of uPA association to uPAR as suggested from SPR experiments [15].

Effects of the aptamers on the binding of pro-uPA to LRP

The K_D for the binding of pro-uPA to LRP-1A is around 10–20 nM [22]. The binding involves interactions of all three domains of uPA with LRP [23]. Using an SPR setup with LRP-1A immobilized on the sensor surface, we examined the ability of the two uPA aptamers to inhibit the binding of pro-uPA to LRP (Fig. 6A and 6B). When passing pro-uPA over the sensor surface after pre-incubation with or without uPA aptamers or a non-relevant control RNA, both of the aptamers, but not the control RNA, were able to dose-dependently inhibit the binding of pro-uPA to LRP-1A.

Effects of the aptamers on the pro-uPA-mediated uPAR-vitronectin binding

Upanap-126 was previously found to inhibit binding of the pro-uPA:uPAR complex to vitronectin in an ELISA setup and pro-uPA:uPAR complex-induced lamellipodia formation in cultured cells [15]. The affinity of vitronectin to uPAR is regulated by a uPA-induced conformational change in uPAR [24]. We observe that unlike upanap-126, upanap-12 does not inhibit the binding of pro-uPA:uPAR complexes to vitronectin coupled to a SPR sensor surface (Fig. 6C and 6D). This observation is in excellent agreement with upanap-12 not being able to bind pro-uPA:uPAR complexes (S3 Fig.).

Effects of the aptamers on the inhibitory activity of PAI-1 towards uPA

Both uPA aptamers were previously demonstrated to bind the zymogen as well as the active form of uPA [14, 15]. Accordingly, we investigated if the two uPA aptamers interfere with the uPA-PAI-1 reaction. A 100-fold excess of either upanap-126 or upanap-12 over uPA did not affect the reaction between uPA and PAI-1 compared with control RNA (data not shown). This observation is in agreement with the fact that the aptamers do not inhibit uPA-mediated plasminogen activation either, which also requires access to the active site [14, 15].

Small-angle X-ray scattering (SAXS) analysis of upanap-12.49 and the aptamer:pro-uPA complex

SAXS analysis was already applied to characterize the overall shape of full-length pro-uPA and active uPA [25]. In order to obtain low-resolution structural information regarding the relative

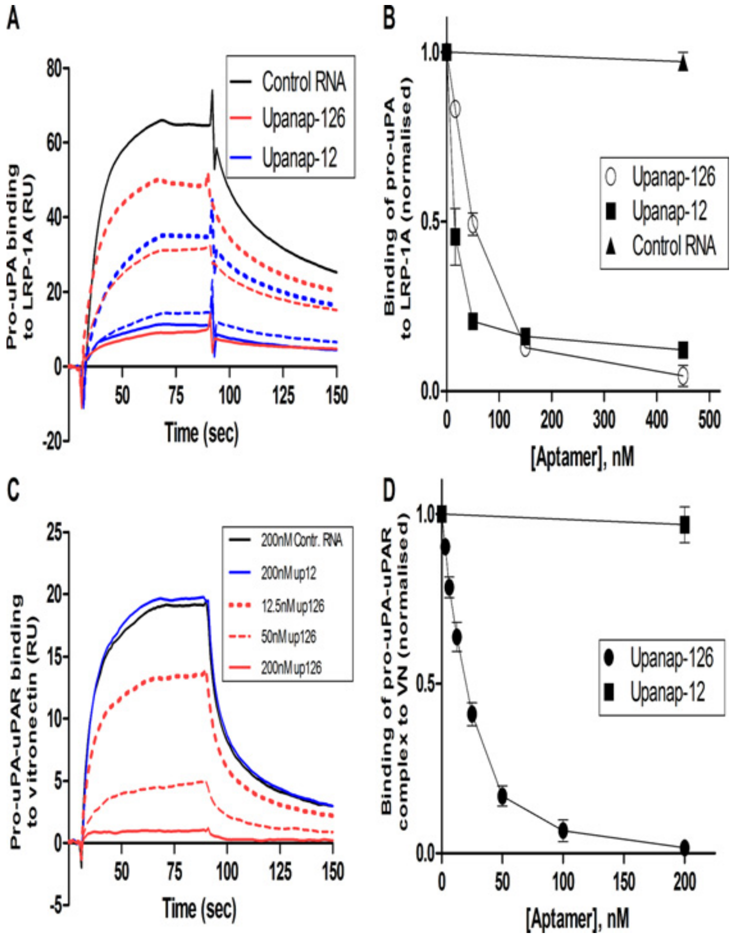

Fig 6. Aptamer inhibition of the binding of pro-uPA to LRP and of the binding of pro-uPA:uPAR complexes to vitronectin. (**A**) SPR sensorgram showing the binding of samples containing 25 nM pro-uPA to LRP immobilized on the sensor surface. The pro-uPA was pre-incubated with control RNA (black), upanap-12 (blue), or upanap-126 (red), as indicated. Solid line (150 nM RNA), broken line (50 nM RNA) and dotted line (17 nM) RNA. No significant effect was observed for 200 nM control RNA relative to no addition (data not shown). (**B**) The number of RU bound to LRP on the sensor surface with 25 nM pro-uPA and varying aptamer concentrations was normalized to the amount of RU bound in the absence of aptamer and plotted versus the aptamer concentration. (**C**) SPR sensorgram showing the binding of samples of 10 nM pro-uPA:uPAR complex to monomeric vitronectin immobilized on the sensor surface. The pro-uPA:uPAR was pre-incubated with RNA as indicated. (**D**) The number of RU bound to vitronectin on the sensor surface with 10 nM pro-uPA:uPAR complex and varying aptamer concentrations was normalized to the amount of RU bound in the absence of aptamer and plotted versus the aptamer concentration. No significant effect was observed with 200 nM control RNA (omitted in the figure). Data represent the average of three replicates.

doi:10.1371/journal.pone.0119207.g006

position of the aptamer in the quaternary complex with uPA, we performed SAXS analysis of upanap-12 alone and in complex with pro-uPA.

For the analysis, we used the truncated version of the ATF-binding aptamer, upanap-12.49. From the indirect Fourier transformation (IFT) analysis of the SAXS data (S6A Fig.), a 'protein equivalent' molecular weight of ~71 kDa was determined. This corresponds well with the actual molecular weight of ~16 kDa for the aptamer when taking into account the two times higher scattering length density difference per unit mass of RNA relative to protein as can be calculated from typical partial specific volumes, and which makes it effectively seem like the aptamer has about four times the mass (Table 1 and Fig. 7A). Furthermore, the *p(r)* function indicated

Table 1. Data obtained from the SAXS analysis.

Sample	'Protein equivalent' molecular mass [kDa]	Theoretical molecular mass [kDa]	Radius of gyration [Å]	D_{max} [Å]
Upanap-12.49	71 ± 7 (~18)[a]	16	26.7 ± 1.0	90 ± 5
pro-uPA + upanap-12.49	93 ± 10	66 (114)[b]	34.2 ± 0.2	110 ± 5

[a]Corrected for the two-fold higher scattering length density difference per unit mass of RNA as compared to a protein sample to allow comparison to theoretical molecular mass.

[b]Calculated 'protein equivalent' mass including pro-uPA (~50 kDa) and 4 times the mass of the RNA (see [a]) to allow direct comparison with the molecular mass of the complex.

doi:10.1371/journal.pone.0119207.t001

that the aptamer has an elongated shape, as the maximum of the curve is shifted to the left (Fig. 7A, insert). We subsequently determined the low-resolution shape of upanap-12.49 and the average *ab initio* model illustrates that the aptamer adopts a straight rod-like structure in solution (Fig. 7B and S6B). Using back calculation, the predicted 3D stem loop structure of upanap-12.49, obtained by computational approaches (iFoldRNA) with the best fit to the experimental data was identified having a χ^2-value of 1.60 (Fig. 7A). By visual inspection, the 3D model agrees well with the *ab initio* shape (Fig. 7B).

The SAXS data for pro-uPA alone obtained in this study was comparable to that previously determined (S6C Fig.). Fig. 7C shows the average ab initio model for pro-uPA superimposed

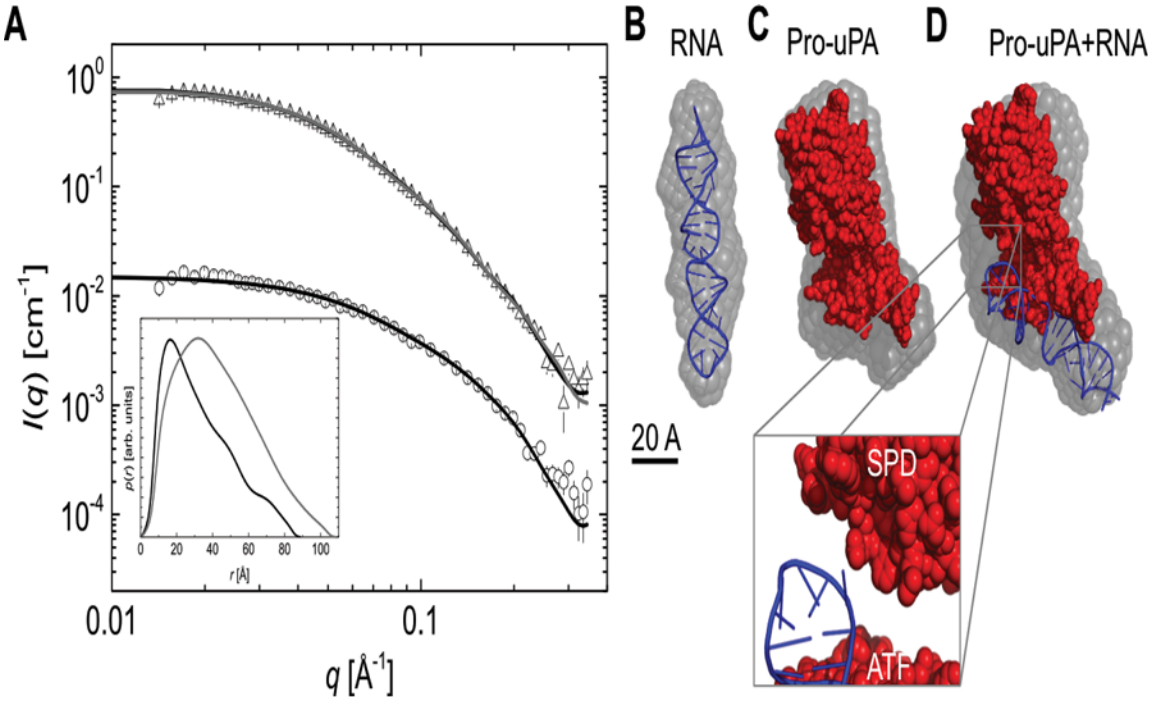

Fig 7. SAXS analysis of upanap-12.49 alone and the complex of upanap-12.49 and pro-uPA. (**A**) Scattering data obtained for free upanap-12.49 (open circles) and the upanap-12.49:pro-uPA complex (open triangels) with their corresponding model fits (black line). The upanap-12.49 data is shown with the CRYSOL fit and the complex with the SASREF fit. The scattering data for the complex is rescaled with a scale factor of 10 to improve visualization of the data. The insert shows the pair distribution functions, p(r), for upanap-12.49 (black) and the aptamer:pro-uPA complex obtained from the IFT of the scattering data. (**B**) Average *ab initio* model for free upanap-12.49 (semitransparent gray) with the best RNA fitting model (blue) superimposed using SUBCOMB alignment [26]. (**C**) Average *ab initio* model for pro-uPA (semitransparent gray) with the previously published structural model of pro-uPA superimposed (red) [25]. (**D**) Average *ab initio* model for the upanap-12.49:pro-uPA complex (semitransparent gray) with the best rigid body model of the upanap-12.49:pro-uPA complex superimposed.

doi:10.1371/journal.pone.0119207.g007

with the previously published structural model of the full-length protein [25]. With SAXS models for upanap-12.49 and pro-uPA as separate entities, we embarked on building a model of the aptamer:pro-uPA complex based on the SAXS data for the complex. From the SAXS data, a 'protein equivalent' molecular weight of ~93 kDa was obtained from the IFT analysis (S6A Fig.), corresponding to a 1:1 complex of expected apparent ~114 kDa when adjusted for the higher scattering length of RNA (Table 1 and Fig. 7A). The *p(r)* function indicated an overall elongated shape for the complex (Fig. 7A, insert), which was generated once again using *ab initio* modeling (Figs. 7D and S6B). As we were interested in the intermolecular arrangement of the molecules, we decided to fit the predicted 3D aptamer model and the pro-uPA SAXS model into the low-resolution shape of the complex (SAXS data) using rigid-body modeling guided by the biochemical data. The generated solutions could be sorted into two subpopulations. The fit of one representative solution is shown in Fig. 7A as all fit equally well to the SAXS data (χ^2 of 1.8 and 1.9 for the most representative models in the two subpopulations, respectively). Both followed the same overall binding pattern and could not be distinguished based on the low-resolution of the SAXS data and the potential rotational freedom of the ATF relative to the catalytic domain (data not shown). A representative solution for these two pools overlay well with the *ab initio* shape of the complex (Fig. 7D). In all solutions, the elongated shape of the aptamer brings it into close proximity to the serine protease domain of uPA when fitted into the SAXS envelope (zoom Fig. 7D). Therefore, under the assumption that the binding of upanap-12 to pro-uPA does not lead to larger conformational changes in the catalytic domain, ATF and/or the aptamer, the best low-resolution rigid-body models based on SAXS data suggest that the aptamer could sterically hinder the access of plasmin to the Lys15-Ile16 bond and hence inhibit pro-uPA activation.

Discussion

Similarly to many other serine proteases, uPA is a modular protein with multi-functional properties. In the present study, we report that two aptamers with different topologic target sites on uPA (upanap-12 binding the ATF and upanap-126 binding the catalytic domain) exert functional pleiotropy and display mutually overlapping inhibitory profiles. We investigated the inhibitory repertoire of these uPA specific aptamers in detail and identified the binding areas of the aptamers by site-directed mutagenesis. Furthermore, using SAXS, we construct a low-resolution structural model of the complex between pro-uPA and a truncated version of the ATF-binding aptamer (upanap-12.49). Table 2 and Fig. 8 summarize the inhibitory profiles of the two aptamers. Our results provide interesting insights into the relationship between aptamer binding sites and their functional activities.

The binding site of the catalytic domain-binding aptamer upanap-126

A major reduction in the affinity of upanap-126 to pro-uPA was observed in the case of the mutants Y284/127A and R391/231A. A smaller reduction in affinity was observed with the mutants R323/166A and K338/179A. All four mutated residues are located near the C-terminal α-helix in the catalytic domain. This localization of the binding site is in agreement with the original selection of the aptamer being driven by the purified catalytic domain in its zymogen form as the bait. Most aptamers are highly specific for their targets and only in a few cases they bind orthologous proteins from other species as observed with FIX and neutrophil elastase aptamers [3]. Upanap-126 is specific for human uPA in the sense that it has no measurable affinity to mouse uPA and the other predominant human plasminogen activator, tPA [15]. This could, at least partly, be governed by the importance of the loop region containing Tyr284/127 for

Table 2. Summary of effects of uPA-binding aptamers on pro-uPA and uPA functions.

Type of uPA activity/interaction	Effect of upanap-126	Effect of upanap-12
uPA catalytic activity (peptidic substrate)	No effect [15]	No effect [14]
uPA catatalytic activity (plasminogen)	No effect [15]	No effect [14]
uPA—PAI-1 reaction	No effect	No effect
Plasmin-catalyzed pro-uPA activation	Inhibition	Inhibition
Plasmin-catalyzed activation of uPAR-bound pro-uPA	Inhibition	No effect
Pro-uPA—uPAR binding	Inhibition	Inhibition
Pro-uPA—LRP-1A binding	Inhibition	Inhibition
Binding of pro-uPA—uPAR complex to VN	Inhibition	No effect

Each type of molecular interaction of uPA is listed in the first column. In the second and the third column, the observed effects of upanap-126 (the catalytic domain-binding aptamer) and upanap-12 (the ATF-binding aptamer) on the molecular interactions are reviewed, respectively.

doi:10.1371/journal.pone.0119207.t002

aptamer binding, which is not conserved in tPA or mouse uPA. Arg391/231, on the contrary, is in a region conserved among many trypsin-like serine proteases.

The pleiotropic effects of upanap-126 on uPA function

In cell culture experiments upanap-126 interferes with uPA:uPAR-mediated lamellipodia formation and cell surface-dependent plasminogen activation [15]. Furthermore, upanap-126 interferes with tumor cell intravasation and invasion in chicken embryo models of cancer [15]. However, there is no evidence that the upanap-126-binding region is directly involved in any natural ligand interactions or activities of uPA. Neither in terms of the catalytic activity of uPA towards small peptidic substrates or plasminogen, the reaction with PAI-1, the plasmin-catalyzed activation of pro-uPA, the binding of uPA to uPAR, the binding of uPA to LRP, nor the binding of the uPA:uPAR complex to vitronectin. It was therefore surprising to find that upanap-126 is an inhibitor of several of uPA′s functions *in vitro* and *in vivo*. The large impact of the Y284/127A and R391/231A mutations on aptamer binding and interference with molecular interactions of uPA demonstrates the specific nature of the uPA-aptamer interaction and rules out the possibility that the observed effects of upanap-126 are due to non-specific binding independent of the identified binding site. Instead, the large size of the 79 nucleotide aptamer most likely facilitates long range steric interference at sites distant from the identified binding region (Table 2 and Fig. 8). Although we would like to further evaluate the size of the aptamer relative to its functions, we have so far not been able to produce shorter variants of upanap-126. Interestingly, the binding site of upanap-126 in pro-uPA corresponds to the binding site of the exosite II-binding 2′-F-Y RNA aptamer Toggle25 in thrombin [7, 27]. Inspecting the crystal structure of thrombin in complex with the truncated 25 nucleotide aptamer variant Toggle-25t [7], a three times larger aptamer such as upanap-126 could easily extend to interfere with plasmin access to the activation site in the pro-uPA catalytic domain. Alternatively, the aptamer could interfere with an as yet unknown exosite interaction for plasmin. The effect of Toggle-25 on zymogen activation has not been determined but crystal structures of thrombin with and without aptamer does not indicate structural or allosteric changes in the serine protease domain upon aptamer binding [7]. In the case of upanap-126 (and upanap-12) uPA binding does not affect the peptidolytic activity of uPA or the uPA—PAI-1 reaction (Table 2 and Fig. 8), suggesting no substantial allosteric changes in the protease domain either.

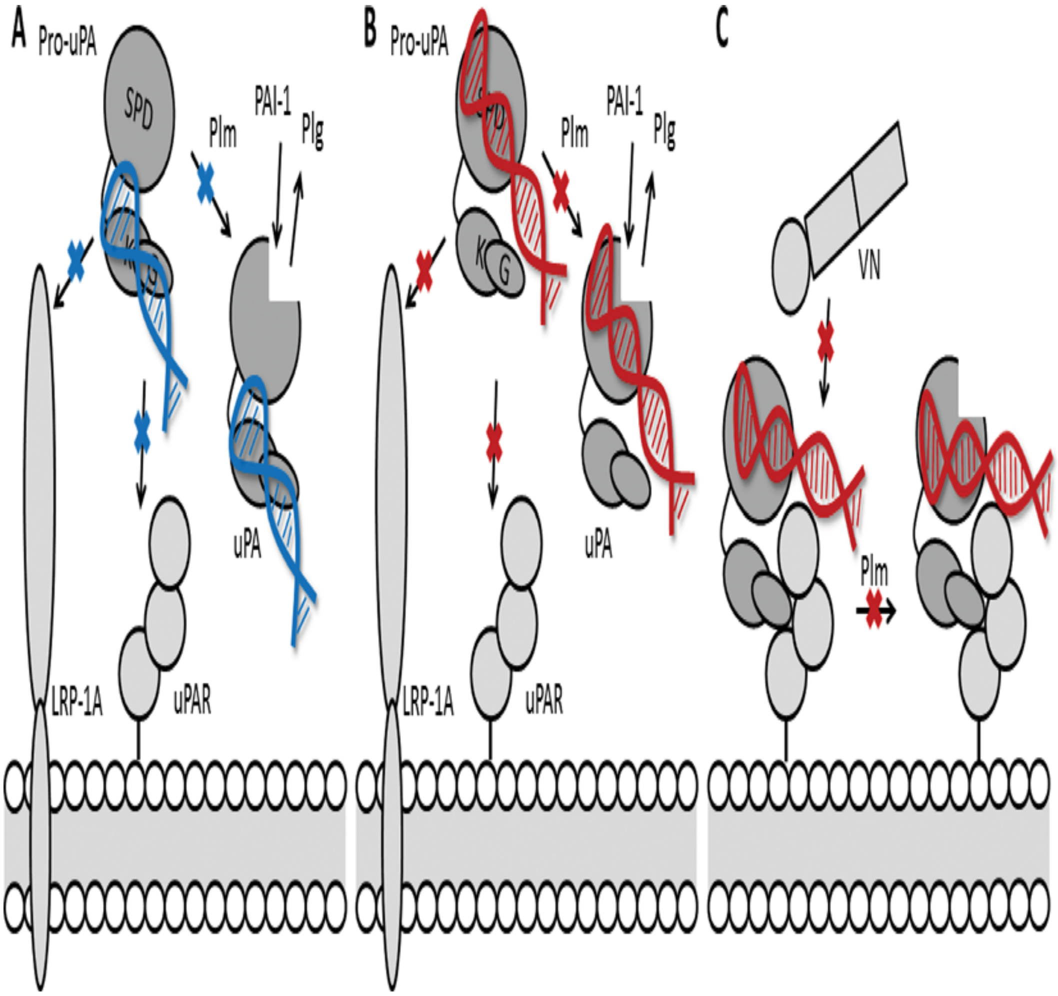

Fig 8. Overview of uPA aptamer-mediated effects on uPA functions. (**A**) The ATF-binding aptamer (upanap-12, blue) binds to a composite site in the kringle and growth factor domain. The domain organization of pro-uPA as well as the size and position of the aptamer in the complex allows it to interfere with plasmin-catalyzed pro-uPA activation and interactions of pro-uPA with uPAR and LRP-1A. (**B**) The aptamer upanap-126 (red) binds to the catalytic domain of pro-uPA positioning it to interfere with pro-uPA activation as well as pro-uPA interaction with uPAR and LRP-1A. (**C**) The interdomain organization of pro-uPA, possibly in combination with some flexibility in the linker region between the catalytic domain and the kringle domain, allows upanap-126 to bind pro-uPA concomitantly with uPAR. Upanap-126 is therefore able to inhibit the binding of pro-uPA:uPAR complexes to vitronectin in addition to plasmin-catalyzed activation of uPAR-bound pro-uPA.

doi:10.1371/journal.pone.0119207.g008

The exact orientation of the ATF relative to the catalytic domain in the three-dimensional structure of pro-uPA is not known. However, the upanap-126 binding site is close to the attachment site of the interdomain linker (C148/1) connecting the kringle domain to the catalytic domain. Hence, the aptamer could be located close to the ATF (Fig. 8), enabling the observed sterical inhibition of pro-uPA β-hairpin burial in uPAR [28]. Still, minor flexibility in the interdomain linker or the aptamer would allow upanap-126 to bind pro-uPA concomitantly with uPAR and disturb the uPAR—vitronectin interaction site located just ~10–15 Å from the ATF, as revealed by the ATF:uPAR:SMB structure [28]. Concerning the pro-uPA—LRP inhibitory activity, site-directed mutagenesis implicated all three domains of uPA in the binding of uPA:PAI-1 complexes to endocytosis receptors [23]. In the catalytic domain, the 37- and 60s-loop were found to be important for LRP binding, but as these loops are localized on the

opposite side of the catalytic domain as compared with the upanap-126 binding region, it would suggest that the aptamer probably interferes with ATF—LRP interactions.

Most of the experiments in the present study were performed with pro-uPA. Although, upanap-126 binds both pro-uPA and uPA, we found previously that upanap-126 interferes better with the interaction of pro-uPA to uPAR than uPA to uPAR [15]. It is currently not known if this is the result of a reduced affinity of upanap-126 for uPA or due to the increased interdomain flexibility accompanying pro-uPA activation [25]. However, some differences in inhibition of uPA relative to pro-uPA may therefore also be observed concerning the effect of upanap-126 towards uPA:uPAR complex binding to vitronectin and uPA binding to LRP.

The binding site of the ATF-binding aptamer upanap-12

Mutation of several residues in the kringle domain (Lys46, Lys48, Lys61 and Lys98) and the β-hairpin of the growth factor domain (Asn22, Lys23, Tyr24, and Trp30) was found to have a measurable effect on upanap-12 binding to pro-uPA, while no interactions with the catalytic domain were suggested by the mutagenesis analysis. The residues of the β-hairpin are all positioned so that the side-chains point towards the solvent from the same face of the β-sheet [18]. The mutation W30A had the largest effect on upanap-12 binding to pro-uPA and protected pro-uPA efficiently from the inhibitory effect of upanap-12 towards plasmin-catalyzed activation. The side-chain of Trp30 is at the center of the upanap-12 binding site, highly surface-exposed and probably optimally oriented for extensive interaction with the aptamer, unlike for example Asn22, which is to some extent buried. Particularly Lys23, Tyr24, Phe25, Ile28, and Trp30 have been determined to be important for human uPA—uPAR binding [19, 21]. Thus, the upanap-12 binding site overlaps with the uPAR binding site. In addition, the hotspot nature of Trp30 provides an explanation for the species selectivity of the aptamer, as Trp30 in human uPA is replaced by Arg31 in murine uPA, a difference of high importance for the species selectivity observed in the uPA—uPAR interaction [19, 21]. Previously, we showed that upanap-12 does not bind to a variant of uPA lacking the growth factor domain [14]. However, the aptamer was also unable to compete out the binding of this domain alone to uPAR (Fig. 5B). Therefore, the results confirm that the binding of the aptamer appears to require a surface composed of residues from both the kringle and growth factor domain.

The multiple effects of upanap-12 on the molecular interactions of uPA (Table 2 and Fig. 8)

The overlap between the binding sites of upanap-12 and uPAR on uPA readily explains how upanap-12 is able to inhibit pro-uPA binding to uPAR-expressing cells, uPAR-dependent endocytosis of the uPA:PAI-1 complex and cell surface-associated plasminogen activation initiated by exogenous addition of pro-uPA [14]. It also readily explains why upanap-12 does not interfere with the catalytic activity of uPA, with the uPA—PAI-1 reaction, or with the molecular interactions of the pro-uPA:uPAR complex with vitronectin. Also, the inhibition of the pro-uPA—LRP binding by upanap-12 is in agreement with both the kringle and growth factor domains having been implicated in LRP binding [23]. However, it was surprising that the aptamer is able to interfere with plasmin-catalyzed pro-uPA activation, as the cleavage site is localized in the catalytic domain, presumably at a distance from the upanap-12 binding site. Even truncating the aptamer from the full-length 79 nucleotides to 49 nucleotides (upanap-12.49) or 33 nucleotides (upanap-12.33) did not reduce this inhibitory activity of the aptamer. Currently, however, the molecular details concerning plasmin recognition of the activation domain of pro-uPA are unknown.

The SAXS structure of upanap-12.49 and the upanap-12.49-pro-uPA complex

To the best of our knowledge, no serine protease-binding aptamers have previously been investigated by the SAXS technique. SAXS is able to provide low-resolution information on shape and dimension of homogeneous molecules in solution. In particular, SAXS is an interesting tool for studying the overall shape of multi-domain proteins, for which other structural approaches may fail. In the case of pro-uPA, the full-length structure has only been determined by SAXS [25]. Here, we used SAXS to determine the shape and dimensions of upanap-12.49 and upanap-12.49 in complex with pro-uPA. Upanap-12.49 is well-described by a rod-like shape in solution, in good agreement with the elongated stem-loop structure proposed by computational methods. Helical segments are also in accordance with regions of covariance when comparing upanap-12 related sequences, while the hairpin and internal loop sequences are highly conserved indicating potential areas of direct contact with uPA [14]. Interestingly, the SAXS analysis shows that even though the molecular mass of upanap-12.49 is ~3 times less than that of pro-uPA (~50 kDa), the estimated length of the folded 49 nucleotide aptamer determined here (~90 Å) would still allow it to span almost the entire length of pro-uPA (~110 Å) (Table 1) [25]. In addition, the SAXS result demonstrates the relatively low compactness of a folded polynucleotide chain compared to a polypeptide of similar molecular weight (radius of gyration ~27 Å and 30 Å for the aptamer and pro-uPA, respectively) (Table 1). Therefore, it is not surprising that the two uPA-binding aptamers are able to interfere extensively with uPA's molecular interactions. The estimated shape of the aptamer:pro-uPA complex, relative to those of each of the two molecules separately, suggests that upanap-12.49, rather than protruding away from pro-uPA into the solvent, packs extensively against the ATF and the interdomain region between the catalytic domain and the ATF. The SAXS analysis suggests that the pro-uPA-bound aptamer is in close proximity to the catalytic domain, thereby allowing for steric interference with the access of plasmin to the cleavage site.

Conclusion

Here, we investigated the molecular mechanisms behind the pleiotropic effects of two uPA-binding aptamers with binding sites in separate regions of the protease. Using mutational and functional analysis, we mapped the aptamer binding sites and demonstrate the high specificity in binding and functionality of the aptamers. Our study shows that aptamers may interfere with the binding of ligands at sites considered remote from the aptamer binding site. SAXS structural analysis suggests that this may be a combination of two structural effects. First, the size and shape of an aptamer may allow it to extend and interfere sterically with binding events in the protein distant from its own binding site. Second, apparent distant functional sites of the protein may be brought into proximity of the aptamer binding site by the overall domain organization of the protein target.

Experimental Procedures

Proteins and RNA

Recombinant purified human pro-uPA was generously provided by Abbott Laboratories (Abbott Park). Recombinant PAI-1 was prepared as described before [29]. The ATF was prepared by proteolytic cleavage of active uPA (Wakamoto) [30]. Human uPAR, and pro-uPA mutants with single alanine substitutions in the ATF were prepared as described [20, 21], by expression in Drosophila S2 cells. The GFD^{4-43} domain was excised from recombinant pro-uPA by Glu-C digestion and purified by size exclusion chromatography [31]. Pro-uPA mutants with alanine

substitutions in the catalytic domain were expressed in human embryonic kidney 293 (HEK293) 6E suspension cells after cloning of the cDNA encoding full-length uPA into the pcDNA3.1 vector followed by site-directed mutagenesis. HEK293 6E cells were cultured in F17 media containing 4 mM L-glutamine, 0.1% FP68, 100 units/mL penicillin, 100 units/mL streptomycin and 25 ug/mL G418 (Life Technologies). Transfection was carried out by pre-incubating 22 μg linear polyethyleneimine (PEI) and 11 μg of vector in 1.1 mL PBS for 15 minutes, and then adding the solution to 10 mL of culture with a density of 10^6 cells/mL. Conditioned media were harvested 5 days later and the concentration of pro-uPA in the media estimated by SPR, using an anti-uPA antibody mAb-6 setup (see SPR analysis below), comparing the binding response to a calibration curve of purified pro-uPA. No pro-uPA was detected in mock-transfected media. No enzymatic activity was observed for any pro-uPA variant, using 0.5 mM of the uPA chromogenic substrate L-Pyroglutamyl-glycyl-L-arginine-p-Nitroaniline hydrochloride (CS-61(44); Aniara) over 3 hours at 37°C in HEPES-buffered saline (HBS; 20 mM HEPES, 140 mM NaCl, pH 7.4) containing 2 mM $MgCl_2$, 0.1% BSA, confirming that variants were in the zymogen form. 2′-F-Y RNA aptamers were produced and purified as described [14, 15]. Briefly, RNA was transcribed from dsDNA transcription templates containing a T7 promotor followed by the aptamer sequence in reactions of 80 mM HEPES (pH 7.5), 30 mM DTT, 25 mM MgCl2, 2 mM spermidine-HCl, 2.5 mM ATP and GTP (Thermo Scientific), 2.5 mM 2′-F-dCTP and 2′-F-dUTP (TriLink Biotechnologies), 100 μg/mL BSA (Thermo Scientific), 0.5–1 μM dsDNA template, and 150 μg/mL mutant T7 RNA polymerase Y639F. RNA transcripts were purified by 8% denaturing polyacrylamide gel electrophoresis (National Diagnostics), retrieved by passive elution followed by ethanol precipitation. Aptamer sequences can be found in S1 Table.

Localization of binding sites for uPA-binding aptamers by SPR analysis

Analysis was performed with a Biacore T200 (GE Healthcare). An anti-uPA antibody mAb-6 [32] was coupled onto an EDC/NHS-activated CM5 sensor surface to a level of 5000 RU, using a buffer of 10 mM Na acetate pH 5. Pro-uPA variants were captured at levels of 200 RU, followed by recording of the binding level response of 15 nM upanap-126, upanap-12 or a control RNA [14]. RNA samples were prepared in running buffer (HBS, 2 mM $MgCl_2$, 0.1% BSA and 0.005% Tween 20). Sensor surfaces were regenerated with 10 mM glycine-HCl (pH 2.5) containing 0.5 M NaCl. The number of response units (RU) of bound RNA was divided by the number of RU of captured pro-uPA, in order to identify mutations reducing RNA binding.

SPR analysis of uPA aptamer interference with the binding of uPA variants to uPAR

For studying aptamer competition with the binding of pro-uPA, ATF or GFD to uPAR, uPA variants (4 nM) were passed over a sensor surface coupled with 1000 RU of uPAR (using 15 μg/mL uPAR in 10 mM Na acetate, pH 4.5) in the presence of increasing concentrations of aptamer. Regeneration between cycles was accomplished with 10 mM glycin-HCl (pH 2.5), 0.5 M NaCl. The inhibition of pro-uPA variant binding to uPAR by aptamers was determined based on the amount of bound pro-uPA after 80 s sample injections.

Binding of aptamers to the pro-uPA:uPAR complex was investigated using a sensor surface coupled with 5000 RU anti-uPAR antibody R2 [24] (using 50 μg/mL R2 in Na acetate, pH 5). uPAR and then pro-uPA were captured and the binding of 100 nM aptamer monitored. The sensor surface was regenerated with 10 mM glycine-HCl (pH 2.5) containing 0.5 M NaCl.

Aptamer inhibition of plasmin-catalyzed pro-uPA activation

For immunoblotting analyses, purified pro-uPA (100 nM) was pre-incubated with or without 200 nM upanap-126, upanap-12, upanap-12.49, upanap-12.33 or a control RNA sequence used previously [14] for 30 minutes in HBS with 2 mM $MgCl_2$. Then, 2.5 nM plasmin (American Diagnostica) was added to the reaction mixtures (time 0). Samples were taken at different time points, acidified with 30 mM HCl and analyzed by reducing SDS-PAGE and immunoblotting using anti-uPA polyclonal antibody F1609 essentially as described [15].

In chromogenic assays, samples were prepared in HBS, 2 mM $MgCl_2$, 0.1% BSA and 0.005% Tween 20. 2 nM pro-uPA was incubated in the presence or absence of 10 nM uPAR for 20 minutes at room temperature prior to the addition of uPA aptamers or control RNA followed by another 30 minutes of incubation. Plasmin (0.5 nM) was then added to the pro-uPA. After 30 minutes, the plasmin activity was quenched with 250 nM aprotinin. The amount of active uPA generated was observed by the relative rate of cleavage (V_i/V_0) of the uPA substrate CS-61(44) (0.5 mM) and plotted as a function of increasing concentrations of RNA.

The uPA—uPAR interaction and the effect of aptamers in cell culture

U937 cells were maintained in RPMI 1640 medium with L-glutamine, supplemented with 10% fetal calf serum (FCS), 100 units/mL penicillin, and 100 units/mL streptomycin (Life Technologies). Purified pro-uPA was labeled with ^{125}I as described [33]. Samples containing 10^6 U937 cells per mL, 10 pM ^{125}I-pro-uPA and 0–500 nM upanap-126, upanap-12 or control RNA control were prepared in culture medium and incubated for 1 or 24 hours at 4°C. The cells were then pelleted and the amount of radioactivity in the pellet and the supernatant determined. The bound ^{125}I-pro-uPA was divided by total ^{125}I-pro-uPA.

Aptamer interference with the pro-uPA—LRP interaction

Murine LRP, kindly provided by Helle Heibroch Petersen, Novo Nordisk A/S, Måløv, Denmark, was coupled (10 μg/mL in 10 mM glycine-HCl pH 2.8) to a SPR sensor surface to a level of 2500 RU. 25 nM of pro-uPA, pre-incubated with increasing concentrations of RNA aptamer, was passed over the chip and the binding level response recorded after a 60 s injection. The sensor surface was regenerated with 10 mM glycine-HCl (pH 2.5), 0.5 M NaCl.

Binding of pro-uPA:uPAR complexes to vitronectin

Monomeric vitronectin (Molecular Innovations) was immobilized (20 ug/mL in 10 mM Na acetate, pH 4.5) on the surface of an SPR sensor surface. 10 nM of pre-formed pro-uPA-uPAR complex, pre-incubated with increasing concentrations of RNA aptamer, was passed over the chip and the binding level recorded after a 60 s injection. The sensor surface was regenerated using 10 mM glycine-HCl (pH 2.5) supplemented with 0.5 M NaCl.

uPA inhibition by PAI-1

uPA (2 nM) was incubated in the presence or absence of aptamers (200 nM) for 30 min at room temperature. PAI-1 was then added at various concentrations (0-5 nM) and the inhibition of uPA activity monitored over time using the chromogenic uPA substrate CS-61(44) (1.5 mM).

Small-angle X-ray scattering data acquisition

SAXS data sets were collected at 25°C on a laboratory-based pin-hole instrument at Aarhus University, Denmark [34]. All data sets were obtained with samples in HBS with 2 mM $MgCl_2$.

Concentrations of upanap-12.49 and pro-uPA in aptamer alone and aptamer:protease complex samples were 0.3 and 0.9 mg/mL, respectively. Background subtraction and conversion to absolute scale of the data was done with water as a primary standard using the SUPERSAXS program package (CLP Oliveira and JS Pedersen, unpublished).

SAXS data analysis and modeling

The pair distance distribution $p(r)$ function was obtained by performing an indirect Fourier transformation (IFT) of the data implemented in the program **WIFT** [35] (CLP Oliveira and JS Pedersen, unpublished), from which, the maximum particle dimension, D_{max}, and the radius of gyration, R_g, were derived. In addition, the forward scattering $I(q = 0)$, calculated from $p(r)$, allows the calculation of the ('protein equivalent') molecular mass of the investigated sample (whether it is pure protein or an aptamer sample) using an average scattering length density difference per unit mass of protein of 2.0 x 10^{10} cm/g. Low resolution *ab initio* molecular surface envelopes were calculated for upanap-12.49 and upanap-12.49 in complex with pro-uPA using the program DAMMIF [36]. Ten DAMMIF solutions were compared and averaged with DAMAVER [37] resulting in a similarity measure (the average normalized discrepancy, NSD) used to evaluate data quality and whether more than one population of structures dominates the models. It should be noted that the *ab initio* method assumes the same scattering length for all dummy atoms used to construct the low-resolution structural model and has a bias towards compact objects. Thus, structural models for complexes of protein and RNA might have some distortions, as the method attempts to assign more scattering length (*i.e.* to put more dummy atoms) at the position of the RNA. The rigid-body optimization method does not have this problem, as the individual components of the complex are assigned the correct scattering length. The program CRYSOL [38] was used to compare theoretically calculated RNA 3D model outputs from iFoldRNA [39, 40] to the experimental scattering data of the aptamers, and the best solution was chosen for further modeling studies. Rigid-body modeling of the upanap12.49:pro-uPA complex was performed using the selected iFoldRNA model of upanap-12.49 and the previously determined model of pro-uPA by SAXS [25] using the program SASREF [41]. A loose distance constraint between pro-uPA and upanap-12 was applied, corresponding to ¼ of the length (25 Å) of the cylinder-symmetric low-resolution model of the free aptamer, allowing any region of the aptamer to interact with the ATF of pro-uPA as suggested by the biochemical data. The constraint was specified between position Trp30 of the ATF (centered in the binding site) and the symmetry based position 17 of the upanap-12.49. For further information on modeling of protein-RNA complexes in general [42]. All PDB files were visualized in PyMOL (The PyMOL Molecular Graphics System, Version 1.5.0.4 Schrödinger, LLC).

Supporting Information

S1 Table. RNA Aptamer sequences.
(PDF)

S1 Fig. SPR analysis overview of aptamer binding to pro-uPA mutants. Each of the 74 pro-uPA alanine mutants were captured on a SPR sensor surface carrying immobilized kringle-specific anti-uPA antibody mAb-6 at a level of around 200 RU. The binding level achieved after 60 seconds of association of either 15 nM upanap-126 or upanap-12 was subsequently recorded. For each mutant, the exact number of RU of bound aptamer was divided by the number of RU of captured pro-uPA. The resulting number was normalized against the number for pro-uPA wild type. The figure summarizes the results with A-chain mutants (**A**) and B-chain mutants (**B**).
(TIF)

S2 Fig. Catalytic activity of mutants of uPA with reduced binding to upanap-126. Catalytic activity of 5 nM wild type uPA (black squares) or uPA mutants Y284/127A (blue triangles), R323/166A (red spheres), K338/179A (green diamonds) and R391/231A (purple triangles) towards 250 μM of peptidic chromogenic uPA substrate after complete activation (2 hours with 2.5 nM plasmin). Absorbance at 405 nm was monitored over time and did not indicate any major differences between variants in terms of catalytic activity. The results represent one of three similar independent measurements.
(TIF)

S3 Fig. SPR sensorgram for the analysis of the binding of upanap-12 and upanap-126 to uPAR-bound pro-uPA. A sensor surface was coupled with anti-uPAR antibody R2. uPAR was subsequently passed over the sensor surface followed by pro-uPA. The association and dissociation of 100 nM upanap-12 (broken line) and upanap-126 (black line) upon injection is shown.
(TIF)

S4 Fig. SPR analysis of upanap-126 interference with the binding of uPA mutants to uPAR. The SPR sensorgrams (**A**-**C**) show examples of the association and dissociation phases for binding of 2 nM wt pro-uPA (**A**) as well as pro-uPA mutants R391/231A (**B**) or Y284/127A (**C**) to uPAR on the sensor surface (black lines). In each figure coloured lines represent wild type pro-uPA pre-incubated with either 3.13 nM (green), 12.5 nM (blue) or 50 nM (purple) upanap-126 (**A**), or pro-uPA mutants pre-incubated with 200 nM (red) upanap-126 (**B** and **C**, respectively).
(TIF)

S5 Fig. SPR analysis of upanap-12 interference with the binding of uPA mutants to uPAR. 4 nM of pro-uPA, ATF or GFD was passed over a CM5 surface with immobilized uPAR with or without upanap-12. In **A**-**C** the black line represents binding to uPAR of either pro-uPA (**A**), ATF (**B**) or GFD (**C**) alone without RNA. In **A** and **B** the coloured lines represent the binding of pro-uPA or ATF to uPAR after pre-incubation with 0.78 nM (green), 3.13 nM (blue) and 12.5 nM (purple) of upanap-12 (). In **C**, the red line represents binding of GFD to uPAR after pre-incubation with 50 nM of upanap-12.
(TIF)

S6 Fig. Supporting SAXS data. (**A**) SAXS data obtained for the free upanap-12.49 aptamer (open circles) and the aptamer:pro-uPA complex (open triangels) with their corresponding IFT model fits (black line). (**B**) SAXS data obtained for free upanap-12.49 (open circles) and upanap-12.49:pro-uPA complex (open triangels) with their corresponding model fits (black line) for the most representative *ab initio* model. The SAXS data for the complex in panel A and B is rescaled with a scale factor of 10 to improve visualization of the data. (**C**) SAXS data obtained for pro-uPA in the current study (closed circles) compared to pro-uPA from a previous study [25] (open circles). (**D**) Rigid-body models for the upanap-12.49:pro-uPA complex with the full-length pro-uPA structural model in red and the two most representative aptamer12.49 models in green and blue. The semitransparent beads represent the *ab initio* space of the complex after subtraction of the *ab initio* space of uPA, illustrating the residual space for the RNA to occupy.
(TIF)

Author Contributions

Conceived and designed the experiments: DMD PAA. Performed the experiments: DMD CKT KAB MAB KD. Analyzed the data: DMD CKT KAB MAB KD JKJ JSP. Contributed reagents/materials/analysis tools: DMD MP JSP HPS. Wrote the paper: DMD MP MAB JKJ PAA.

References

1. Tuerk C, Gold L. Systematic evolution of ligands by exponential enrichment: RNA ligands to bacteriophage T4 DNA polymerase. Science. 1990; 249(4968):505–10. Epub 1990/08/03. PMID: 2200121
2. Ellington AD, Szostak JW. In vitro selection of RNA molecules that bind specific ligands. Nature. 1990; 346(6287):818–22. Epub 1990/08/30. doi: 10.1038/346818a0 PMID: 1697402
3. Dupont DM, Andersen LM, Botkjaer KA, Andreasen PA. Nucleic acid aptamers against proteases. Curr Med Chem. 2011; 18(27):4139–51. Epub 2011/08/16. doi: BSP/CMC/E-Pub/2011/311[pii] PMID: 21838691
4. Keefe AD, Pai S, Ellington A. Aptamers as therapeutics. Nat Rev Drug Discov. 2010; 9(7):537–50. Epub 2010/07/02. doi: nrd3141 [pii] 10.1038/nrd3141 PMID: 20592747
5. Drag M, Salvesen GS. Emerging principles in protease-based drug discovery. Nat Rev Drug Discov. 2010; 9(9):690–701. Epub 2010/09/03. doi: nrd3053 [pii] 10.1038/nrd3053 PMID: 20811381
6. Bock LC, Griffin LC, Latham JA, Vermaas EH, Toole JJ. Selection of single-stranded DNA molecules that bind and inhibit human thrombin. Nature. 1992; 355(6360):564–6. Epub 1992/02/06. doi: 10.1038/355564a0 PMID: 1741036
7. Long SB, Long MB, White RR, Sullenger BA. Crystal structure of an RNA aptamer bound to thrombin. RNA. 2008; 14(12):2504–12. Epub 2008/10/31. doi: rna.1239308 [pii] 10.1261/rna.1239308 PMID: 18971322
8. Andreasen PA, Kjoller L, Christensen L, Duffy MJ. The urokinase-type plasminogen activator system in cancer metastasis: a review. Int J Cancer. 1997; 72(1):1–22. Epub 1997/07/03. doi: 10.1002/(SICI)1097-0215(19970703)72:1<1::AID-IJC1>3.0.CO;2-Z [pii] PMID: 9212216
9. Carriero MV, Stoppelli MP. The urokinase-type plasminogen activator and the generation of inhibitors of urokinase activity and signaling. Current pharmaceutical design. 2011; 17(19):1944–61. Epub 2011/06/30. PMID: 21711235
10. Wei Y, Waltz DA, Rao N, Drummond RJ, Rosenberg S, Chapman HA. Identification of the urokinase receptor as an adhesion receptor for vitronectin. J Biol Chem. 1994; 269(51):32380–8. Epub 1994/12/23. PMID: 7528215
11. Dupont DM, Madsen JB, Kristensen T, Bodker JS, Blouse GE, Wind T, et al. Biochemical properties of plasminogen activator inhibitor-1. Front Biosci (Landmark Ed). 2009; 14:1337–61. Epub 2009/03/11. doi: 3312[pii] PMID: 19273134
12. Gliemann J, Nykjaer A, Petersen CM, Jorgensen KE, Nielsen M, Andreasen PA, et al. The multiligand alpha 2-macroglobulin receptor/low density lipoprotein receptor-related protein (alpha 2MR/LRP). Binding and endocytosis of fluid phase and membrane-associated ligands. Ann N Y Acad Sci. 1994; 737:20–38. Epub 1994/09/10. PMID: 7944146
13. Harbeck N, Sotlar K, Wuerstlein R, Doisneau-Sixou S. Molecular and protein markers for clinical decision making in breast cancer: Today and tomorrow. Cancer Treat Rev. 2014; 40(3):434–44. Epub 2013/10/22. doi: 10.1016/j.ctrv.2013.09.014 S0305-7372(13)00203-X [pii] PMID: 24138841
14. Dupont DM, Madsen JB, Hartmann RK, Tavitian B, Duconge F, Kjems J, et al. Serum-stable RNA aptamers to urokinase-type plasminogen activator blocking receptor binding. RNA. 2010; 16(12):2360–9. Epub 2010/10/22. doi: rna.2338210 [pii] 10.1261/rna.2338210 PMID: 20962041
15. Botkjaer KA, Deryugina EI, Dupont DM, Gardsvoll H, Bekes EM, Thuesen CK, et al. Targeting tumor cell invasion and dissemination in vivo by an aptamer that inhibits urokinase-type plasminogen activator through a novel multifunctional mechanism. Mol Cancer Res. 2012; 10(12):1532–43. Epub 2012/10/06. doi: 10.1158/1541-7786.MCR-12-0349 1541-7786.MCR-12-0349 [pii] PMID: 23038812
16. Arnold K, Bordoli L, Kopp J, Schwede T. The SWISS-MODEL workspace: a web-based environment for protein structure homology modelling. Bioinformatics. 2006; 22(2):195–201. Epub 2005/11/23. doi: bti770 [pii] 10.1093/bioinformatics/bti770 PMID: 16301204
17. Pjura PE, Lenhoff AM, Leonard SA, Gittis AG. Protein crystallization by design: chymotrypsinogen without precipitants. J Mol Biol. 2000; 300(2):235–9. Epub 2000/06/30. doi: 10.1006/jmbi.2000.3851 S0022-2836(00)93851-8 [pii] PMID: 10873462
18. Barinka C, Parry G, Callahan J, Shaw DE, Kuo A, Bdeir K, et al. Structural basis of interaction between urokinase-type plasminogen activator and its receptor. J Mol Biol. 2006; 363(2):482–95. Epub 2006/09/19. doi: S0022-2836(06)01116-8 [pii] 10.1016/j.jmb.2006.08.063 PMID: 16979660
19. Magdolen V, Rettenberger P, Koppitz M, Goretzki L, Kessler H, Weidle UH, et al. Systematic mutational analysis of the receptor-binding region of the human urokinase-type plasminogen activator. Eur J Biochem. 1996; 237(3):743–51. Epub 1996/05/01. PMID: 8647121
20. Gardsvoll H, Gilquin B, Le Du MH, Menez A, Jorgensen TJ, Ploug M. Characterization of the functional epitope on the urokinase receptor. Complete alanine scanning mutagenesis supplemented by chemical

cross-linking. J Biol Chem. 2006; 281(28):19260–72. Epub 2006/05/05. doi: M513583200 [pii] 10.1074/jbc.M513583200 PMID: 16672229

21. Lin L, Gardsvoll H, Huai Q, Huang M, Ploug M. Structure-based engineering of species selectivity in the interaction between urokinase and its receptor: implication for preclinical cancer therapy. J Biol Chem. 2010; 285(14):10982–92. Epub 2010/02/06. doi: 10.1074/jbc.M109.093492 M109.093492 [pii] PMID: 20133942
22. Nykjaer A, Kjoller L, Cohen RL, Lawrence DA, Garni-Wagner BA, Todd RF 3rd, et al. Regions involved in binding of urokinase-type-1 inhibitor complex and pro-urokinase to the endocytic alpha 2-macroglobulin receptor/low density lipoprotein receptor-related protein. Evidence that the urokinase receptor protects pro-urokinase against binding to the endocytic receptor. J Biol Chem. 1994; 269(41):25668–76. Epub 1994/10/14. PMID: 7929271
23. Skeldal S, Larsen JV, Pedersen KE, Petersen HH, Egelund R, Christensen A, et al. Binding areas of urokinase-type plasminogen activator-plasminogen activator inhibitor-1 complex for endocytosis receptors of the low-density lipoprotein receptor family, determined by site-directed mutagenesis. FEBS J. 2006; 273(22):5143–59. Epub 2006/10/18. doi: EJB5511 [pii] 10.1111/j.1742-4658.2006.05511.x PMID: 17042782
24. Gardsvoll H, Jacobsen B, Kriegbaum MC, Behrendt N, Engelholm L, Ostergaard S, et al. Conformational regulation of urokinase receptor function: impact of receptor occupancy and epitope-mapped monoclonal antibodies on lamellipodia induction. J Biol Chem. 2011; 286(38):33544–56. Epub 2011/07/30. doi: 10.1074/jbc.M111.220087 M111.220087 [pii] PMID: 21799009
25. Behrens MA, Botkjaer KA, Goswami S, Oliveira CL, Jensen JK, Schar CR, et al. Activation of the zymogen to urokinase-type plasminogen activator is associated with increased interdomain flexibility. J Mol Biol. 2011; 411(2):417–29. Epub 2011/06/15. doi: 10.1016/j.jmb.2011.05.026 S0022-2836(11)00576-6 [pii] PMID: 21669207
26. Kozin MB, Svergun DI. Automated matching of high- and low-resolution structural models. J Appl Crystallogr. 2001; 34:33–41. doi: 10.1107/S0021889800014126
27. Jeter ML, Ly LV, Fortenberry YM, Whinna HC, White RR, Rusconi CP, et al. RNA aptamer to thrombin binds anion-binding exosite-2 and alters protease inhibition by heparin-binding serpins. FEBS Lett. 2004; 568(1–3):10–4. Epub 2004/06/16. doi: 10.1016/j.febslet.2004.04.087 S0014579304005769 [pii] PMID: 15196942
28. Huai Q, Zhou A, Lin L, Mazar AP, Parry GC, Callahan J, et al. Crystal structures of two human vitronectin, urokinase and urokinase receptor complexes. Nat Struct Mol Biol. 2008; 15(4):422–3. Epub 2008/04/01. doi: 10.1038/nsmb.1404 nsmb.1404 [pii] PMID: 18376415
29. Dupont DM, Blouse GE, Hansen M, Mathiasen L, Kjelgaard S, Jensen JK, et al. Evidence for a pre-latent form of the serpin plasminogen activator inhibitor-1 with a detached beta-strand 1C. J Biol Chem. 2006; 281(47):36071–81. Epub 2006/10/05. doi: M606851200 [pii] 10.1074/jbc.M606851200 PMID: 17018527
30. Egelund R, Petersen TE, Andreasen PA. A serpin-induced extensive proteolytic susceptibility of urokinase-type plasminogen activator implicates distortion of the proteinase substrate-binding pocket and oxyanion hole in the serpin inhibitory mechanism. Eur J Biochem. 2001; 268(3):673–85. Epub 2001/02/13. doi: ejb1921[pii] PMID: 11168406
31. Ploug M, Rahbek-Nielsen H, Ellis V, Roepstorff P, Dano K. Chemical modification of the urokinase-type plasminogen activator and its receptor using tetranitromethane. Evidence for the involvement of specific tyrosine residues in both molecules during receptor-ligand interaction. Biochemistry. 1995; 34(39):12524–34. Epub 1995/10/03. PMID: 7548000
32. Petersen HH, Hansen M, Schousboe SL, Andreasen PA. Localization of epitopes for monoclonal antibodies to urokinase-type plasminogen activator: relationship between epitope localization and effects of antibodies on molecular interactions of the enzyme. Eur J Biochem. 2001; 268(16):4430–9. Epub 2001/08/15. doi: ejb2365[pii] PMID: 11502203
33. Jensen PH, Christensen EI, Ebbesen P, Gliemann J, Andreasen PA. Lysosomal degradation of receptor-bound urokinase-type plasminogen activator is enhanced by its inhibitors in human trophoblastic choriocarcinoma cells. Cell Regul. 1990; 1(13):1043–56. Epub 1990/12/01. PMID: 1966892
34. Pedersen JS. A flux- and background-optimized version of the NanoSTAR small-angle X-ray scattering camera for solution scattering. J Appl Crystallogr. 2004; 37:369–80. doi: 10.1107/S0021889804004170
35. Pedersen JS, Hansen S, Bauer R. The aggregation behavior of zinc-free insulin studied by small-angle neutron scattering. Eur Biophys J. 1994; 22(6):379–89. Epub 1994/01/01. PMID: 8149922
36. Franke D, Svergun DI. DAMMIF, a program for rapid ab-initio shape determination in small-angle scattering. J Appl Crystallogr. 2009; 42:342–6. doi: 10.1107/S0021889809000338
37. Volkov VV, Svergun DI. Uniqueness of ab initio shape determination in small-angle scattering. J Appl Crystallogr. 2003; 36:860–4. doi: 10.1107/S0021889803000268

38. Svergun D, Barberato C, Koch MHJ. CRYSOL—A program to evaluate x-ray solution scattering of biological macromolecules from atomic coordinates. J Appl Crystallogr. 1995; 28:768–73. doi: 10.1107/S0021889895007047

39. Sharma S, Ding F, Dokholyan NV. iFoldRNA: three-dimensional RNA structure prediction and folding. Bioinformatics. 2008; 24(17):1951–2. Epub 2008/06/27. doi: 10.1093/bioinformatics/btn328 btn328 [pii] PMID: 18579566

40. Ding F, Sharma S, Chalasani P, Demidov VV, Broude NE, Dokholyan NV. Ab initio RNA folding by discrete molecular dynamics: From structure prediction to folding mechanisms. Rna-a Publication of the Rna Society. 2008; 14(6):1164–73. doi: 10.1261/Rna.894608

41. Petoukhov MV, Svergun DI. Global rigid body modeling of macromolecular complexes against small-angle scattering data. Biophys J. 2005; 89(2):1237–50. Epub 2005/06/01. doi: S0006-3495(05)72771-5 [pii] 10.1529/biophysj.105.064154 PMID: 15923225

42. Behrens MA, He Y, Oliveira CL, Andersen GR, Pedersen JS, Nielsen KH. Structural analysis of RNA helicases with small-angle X-ray scattering. Methods Enzymol. 2012; 511:191–212. Epub 2012/06/21. doi: 10.1016/B978-0-12-396546-2.00031-0 B978-0-12-396546-2.00031-0 [pii] PMID: 22713321
